# Cold-Adapted Viral Attenuation (CAVA): Highly Temperature Sensitive Polioviruses as Novel Vaccine Strains for a Next Generation Inactivated Poliovirus Vaccine

**DOI:** 10.1371/journal.ppat.1005483

**Published:** 2016-03-31

**Authors:** Barbara P. Sanders, Isabel de los Rios Oakes, Vladimir van Hoek, Viki Bockstal, Tobias Kamphuis, Taco G. Uil, Yutong Song, Gillian Cooper, Laura E. Crawt, Javier Martín, Roland Zahn, John Lewis, Eckard Wimmer, Jerome H. H. V. Custers, Hanneke Schuitemaker, Jeronimo Cello, Diana Edo-Matas

**Affiliations:** 1 Janssen Infectious Diseases and Vaccines, Pharmaceutical Companies of Johnson and Johnson, Leiden, the Netherlands; 2 Department of Molecular Genetics and Microbiology, Stony Brook University, Stony Brook, New York, United States of America; 3 Division of Virology, National Institute for Biological Standards and Control, Potters Bar, United Kingdom; University of California, Irvine, UNITED STATES

## Abstract

The poliovirus vaccine field is moving towards novel vaccination strategies. Withdrawal of the Oral Poliovirus Vaccine and implementation of the conventional Inactivated Poliovirus Vaccine (cIPV) is imminent. Moreover, replacement of the virulent poliovirus strains currently used for cIPV with attenuated strains is preferred. We generated Cold-Adapted Viral Attenuation (CAVA) poliovirus strains by serial passage at low temperature and subsequent genetic engineering, which contain the capsid sequences of cIPV strains combined with a set of mutations identified during cold-adaptation. These viruses displayed a highly temperature sensitive phenotype with no signs of productive infection at 37°C as visualized by electron microscopy. Furthermore, decreases in infectious titers, viral RNA, and protein levels were measured during infection at 37°C, suggesting a block in the viral replication cycle at RNA replication, protein translation, or earlier. However, at 30°C, they could be propagated to high titers (9.4–9.9 Log_10_TCID_50_/ml) on the PER.C6 cell culture platform. We identified 14 mutations in the IRES and non-structural regions, which in combination induced the temperature sensitive phenotype, also when transferred to the genomes of other wild-type and attenuated polioviruses. The temperature sensitivity translated to complete absence of neurovirulence in CD155 transgenic mice. Attenuation was also confirmed after extended *in vitro* passage at small scale using conditions (MOI, cell density, temperature) anticipated for vaccine production. The inability of CAVA strains to replicate at 37°C makes reversion to a neurovirulent phenotype *in vivo* highly unlikely, therefore, these strains can be considered safe for the manufacture of IPV. The CAVA strains were immunogenic in the Wistar rat potency model for cIPV, inducing high neutralizing antibody titers in a dose-dependent manner in response to D-antigen doses used for cIPV. In combination with the highly productive PER.C6 cell culture platform, the stably attenuated CAVA strains may serve as an attractive low-cost and (bio)safe option for the production of a novel next generation IPV.

## Author Summary

The vaccines that are used to protect against poliovirus infection have been available since the 1950s and have brought the eradication of poliomyelitis to our doorstep. For the post-eradication era, an Inactivated Poliovirus Vaccine (IPV) based on attenuated Sabin strains is recommended, as these strains are currently the only option to move to safer manufacturing of IPV. Here we describe three novel poliovirus strains that cannot replicate at 37°C. Their lack of pathogenicity was confirmed by intracerebral inoculation of susceptible transgenic mice that subsequently did not develop any symptoms of poliomyelitis. The inability to replicate at 37°C is caused by multiple mutations which do not revert to virulence after passage in cells. Furthermore, when used as vaccines, these viruses were capable of inducing a potent immune response in rats. At low temperature (30°C) these viruses showed high productivity on the PER.C6 cell line, which has the potential to significantly reduce costs of goods, as previously shown for conventional poliovirus strains. Taken together, these new strains could contribute to a safe, genetically stable, efficacious and affordable IPV.

## Introduction

There are two vaccines that can effectively protect against poliomyelitis which have been available for more than 60 years and are still used today. The Inactivated Poliovirus Vaccine (IPV), today referred to as conventional (c)IPV, was developed in 1955 by Jonas Salk and contains three formalin inactivated, wild-type and neurovirulent poliovirus strains (Mahoney, MEF-1 and Saukett) [1]. In the 1960s Albert Sabin introduced the second vaccine against poliomyelitis: the Oral Poliovirus Vaccine (OPV), a trivalent formulation of three live, attenuated strains (Sabin 1, 2 and 3) [2,3]. OPV and IPV have dramatically reduced the incidence of poliomyelitis since their introduction; with only 74 wild-type poliomyelitis cases worldwide in 2015, restricted to Afghanistan and Pakistan, eradication of the disease is extremely close [4].

Despite the efficacy of OPV, the Sabin strains have the propensity to revert to neurovirulent form [5]. In OPV vaccinees these reverted neurovirulent strains can cause Vaccine-Associated Paralytic Poliomyelitis (VAPP), and via shedding, circulating Vaccine Derived Polioviruses (cVDPVs) [6] can cause poliomyelitis outbreaks in areas of low vaccination coverage [7]. Therefore the cessation of OPV use in routine immunization and full implementation of vaccination with the safer, but more expensive, IPV is required to enable the final stages of eradication and sustain a polio-free status in the years thereafter [8,9].

However, even if eradication is achieved, immunization against poliomyelitis will remain necessary to maintain a polio-free world [10], as the risk of re-emergence of polioviruses from several potential sources (spills of laboratory stocks [11] or vaccine production facilities [12], undetected viruses in remote locations, long term shedders after OPV vaccination [13], bioterrorism, etc.) will persist [14,15]. To minimize these risks, replacing cIPV, which is made from wild-type (virulent) strains, with an IPV made from attenuated (non-virulent) strains, is an approach actively promoted by the World Health Organization (WHO) and its collaborators [16].

Currently the Sabin strains used in OPV are the preferred candidates to replace the wild-type strains. Sabin-based IPV’s (sIPV) have been recently licensed in Japan [17] and China [18], and several others are currently in development [19]. The local licensure and worldwide efforts for clinical development of sIPV illustrate the potential of this vaccine to replace the widely used cIPV. However, uncertainties that exist with regard to affordability of large scale production [20,21], complexities in sIPV dosing strategy due to differences in antigenic content, and absence of standardization compared to cIPV [22–24], have prompted investment in alternative strategies to develop next generation IPVs. Implementation of modern vaccinology techniques to enable the generation of novel vaccine strains that display desired characteristics such us reduced pathogenicity and an immunogenic profile identical to the well-established cIPV strains has been proposed [25,26].

Our aim was to develop novel attenuated strains for IPV manufacture that can address the biosafety issues of cIPV without altering immunogenicity. Our approach for viral attenuation was to develop strains with impaired growth at physiological temperature (≥37°C) but that are still capable of replication to high infectious titer yields at lower (manufacturing) temperatures. We hypothesized that inability to replicate at 37°C would impede reversion to neurovirulent form, resulting in a non-pathogenic phenotype in the natural host. Cold-adaptation (adaptation to growth at low (<37°C) temperature by serial passage) is often associated with reduced replication (or sensitivity) at higher temperatures (37–40°C)). Historically, cold-adaptation has been frequently used to generate attenuated RNA and DNA viruses (reviewed in [27]), including influenza [28,29], measles [30], and rubella vaccine strains [31]. Cold-adapted polioviruses have also been generated in the past by passage at 23–30°C [32–34]. In general, these attenuated polioviruses were also temperature sensitive, as defined by increased replication at lower temperatures (≤37°C) as compared to growth at higher (~40°C) temperatures, but did not necessarily show a complete loss of replication capacity at 37°C (as is also the case for the Sabin strains [35–37]).

By combining empirical and rational methods of attenuation, we generated temperature sensitive poliovirus strains incapable of replication at physiological temperature, that grow to high titers at 30°C, and that have the antigenic profile of (wild-type) cIPV strains. The strains were obtained via serial passage at low temperature and genetic engineering, ultimately resulting in three Cold-Adapted Viral Attenuation (CAVA) vaccine strains, namely: CAVA-1 Mahoney, CAVA-2 MEF-1 and CAVA-3 Saukett. We characterized the CAVA strains with respect to *in vitro* temperature sensitivity, *in vivo* attenuation and *in vivo* immunogenicity, and we investigated the mechanism of, and mutations responsible for, their phenotype.

## Results

### I. Derivation of highly temperature sensitive and attenuated poliovirus strains incapable of replication at 37°C

**Fig 1:**
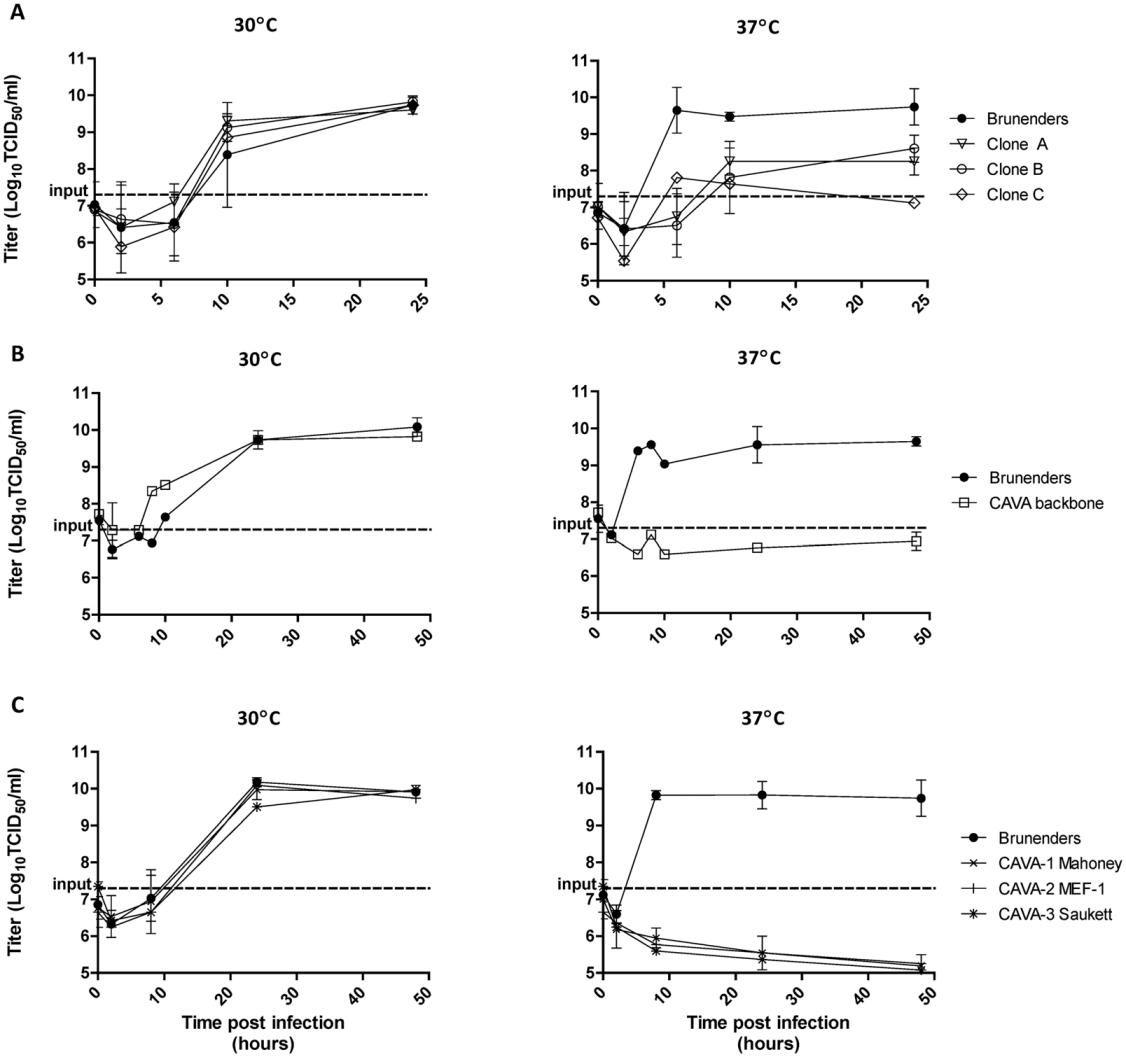
Multiple replication kinetics of infections in PER.C6 cells with a cell density of 10^7^ cells/ml at an MOI of 1–2, at 30°C and 37°C, harvested at 0–48 hours post infection. Panel A) Average and standard deviation of two (n = 2) replication kinetic curves of the Brunenders strain versus 3 selected clones (clone A, B and C) derived after passage with impaired growth at 37°C. Panel B) Average and standard deviation of three (n = 3) independent infections of Brunenders and the CAVA backbone, which contained all mutations from Clones A, B and C combined. Panel C) Average and standard deviation of three (n = 3) independent infections of the Brunenders strain versus the CAVA vaccine strains (CAVA-1 Mahoney, CAVA-2 MEF-1 and CAVA-3 Saukett).

The highly temperature sensitive poliovirus strains were derived from, Brunenders, a Type I partially-attenuated poliovirus [38], by serial passage *in vitro*. The passaging was performed 34 times in PER.C6 cells at low temperature (26–30°C), at low Multiplicity of Infection (MOI = 0.01) and harvested 3–4 days post infection. Adaptation for increased growth at 30°C on PER.C6 cells was observed after 11 and 28 passages, but impairment of growth at 37°C was not detected (S1 Fig). Upon clonal selection, where approximately 1000 clones were screened for infectivity (Cytopathic Effect, CPE) at 30 and 37°C, three clones showed delayed replication at 37°C (1–3 days later) as compared to 30°C. This impaired growth at 37°C was confirmed by comparing growth kinetics in suspension PER.C6 cell cultures at both temperatures (Fig 1A). The three clones showed slower replication rates and a 100- to 1000-fold reduction in maximum titer as compared to the parental Brunenders strain at 37°C, and faster growth at 30°C (Fig 1A) which indicates an adaptation to lower temperature.

**Fig 2:**
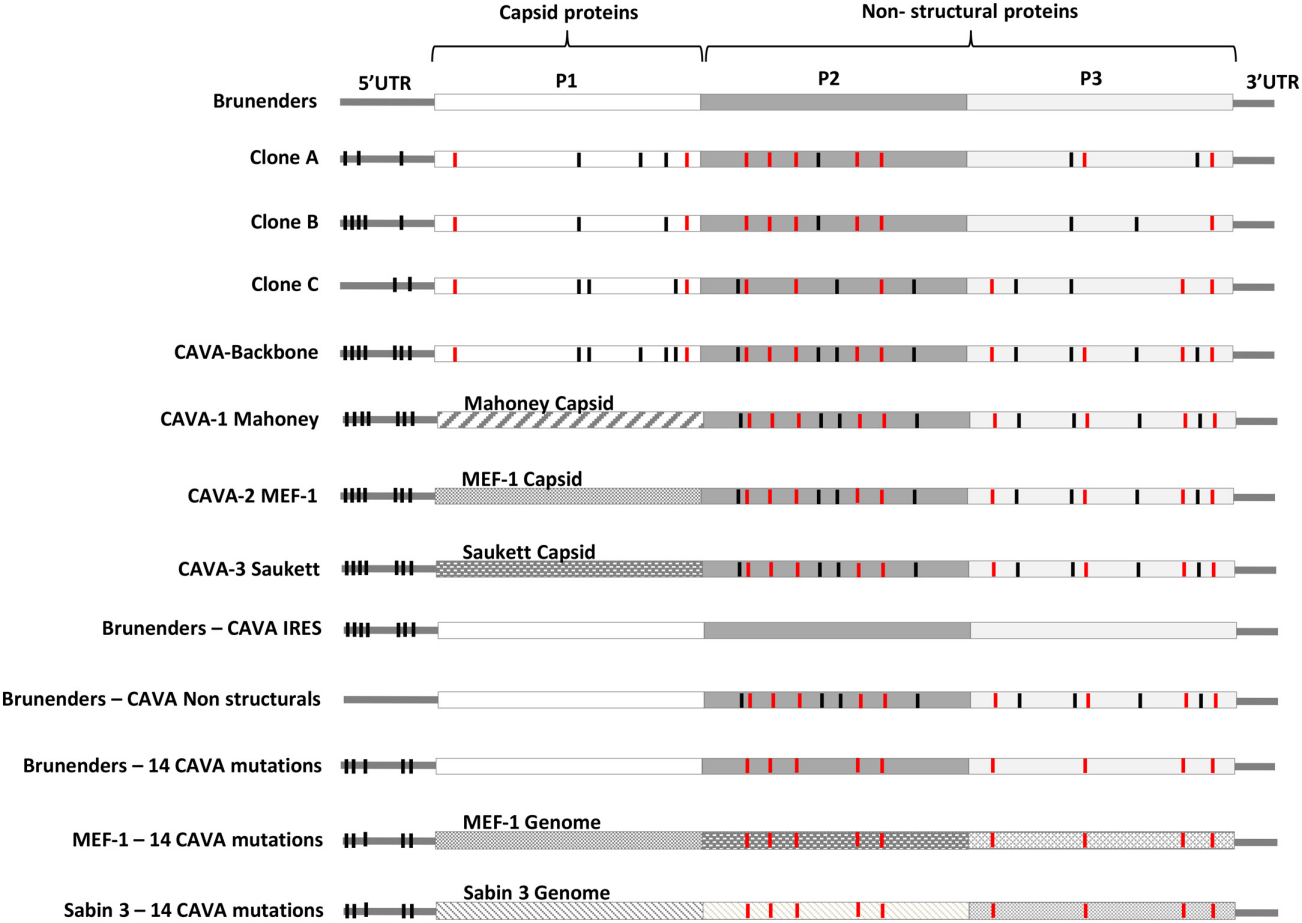
Schematic overview of the viruses described here and their incorporated mutations. Black vertical lines represent the synonymous CAVA mutations whilst red vertical lines represent non-synonymous CAVA mutations, dispersed over the poliovirus genome; a detailed description of the individual mutations is given in S1 Table. 5’UTR = 5’ Untranslated Region, 3’UTR = 3’Untranslated Region.

Each clone had 18 nucleotide mutations (either shared or unique) with respect to the parental Brunenders strain. Overall, 31 distinct mutations were detected across the three different clones (see Fig 2 for a schematic representation of all viruses and S1 Table for details regarding the specific mutations).

All 31 mutations were cloned into the Brunenders cDNA plasmid and transfection of the resulting *in vitro* transcribed RNA in PER.C6 cells resulted in the rescue of the CAVA backbone virus. Remarkably, the combination of 31 mutations was not lethal and viable virus was rescued at 30°C. However, at 37°C, this CAVA backbone virus was incapable of replication (defined as no increase of infectious units (TCID_50_) over a 2 day period). Virus replication at 30°C was unaffected when compared to the Brunenders parental strain (Fig 1B). Temperature sensitivity of the CAVA backbone virus was confirmed when infections were left up to 14 days at 37°C, in multiple cell lines (Vero, L20B, Hela, SK-N-MC, Hek293), and its neuroattenuation *in vivo* was demonstrated (S2 Table) in CD155 transgenic mice, a susceptible model for poliovirus neurovirulence [39].

To generate CAVA-IPV vaccine strains for poliovirus serotypes 1, 2 and 3, the capsid sequence of the CAVA backbone was replaced by the capsid sequences of each of the three cIPV strains, to mimic their antigenic profiles. This resulted in three new synthetically-derived viruses named CAVA-1 Mahoney, CAVA-2 MEF-1 and CAVA-3 Saukett. The remainder of the genome maintained 24 of the CAVA mutations spread over the 5’ Untranslated Region (5’UTR) and Non-Structural proteins (see Fig 2). As was observed with the CAVA-backbone, the three CAVA vaccine strains showed no replication at 37°C, whilst growth kinetics and maximum yields at 30°C were similar to the parental Brunenders strain (Fig 1C).

**Fig 3:**
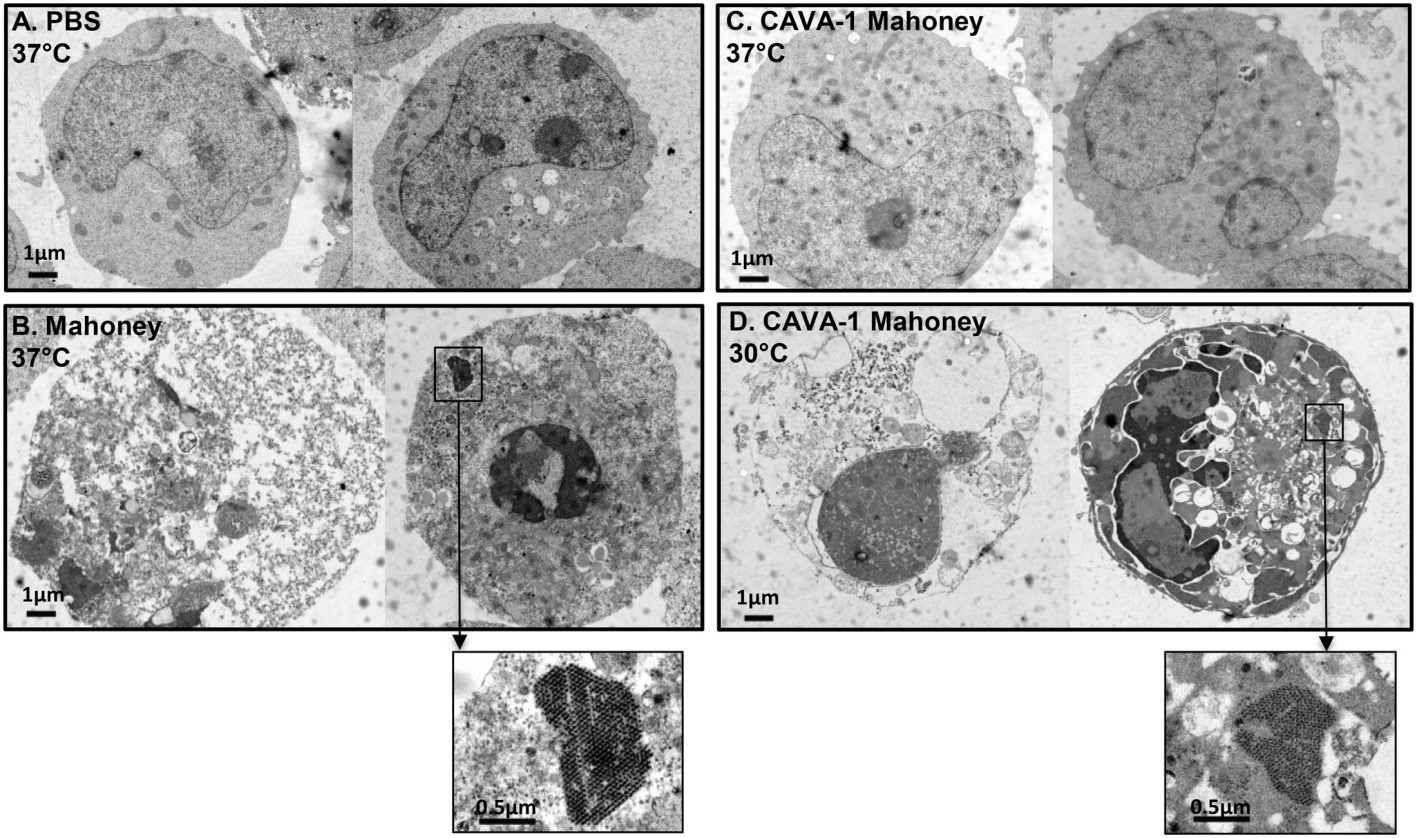
Electron micrographs of two representative cells per panel after infection of suspension PER.C6 cells with a cell density of 10^7^ cells/ml at an MOI of 1, harvested between 24–48 hours post infection. Panel A) PBS (Mock) infected cells, Panel B) Mahoney infection at 37°C, Panel C) CAVA-1 Mahoney at 37°C, Panel D) CAVA-1 Mahoney at 30°C.

To visualize signs of infection, PER.C6 cells were infected with CAVA-1 Mahoney at 37°C and 30°C, at an MOI of 1 and crude harvests were taken 24–48 hours post infection for examination by electron microscopy (EM). PER.C6 cells were infected using the same conditions with either wild-type Mahoney or PBS (mock) at 37°C as positive and negative controls, respectively. Fig 3 depicts representative cells from the inoculated cell cultures; CAVA-1 Mahoney at 30°C resembled the wild-type Mahoney PV infection at 37°C with cells being either dead or dark and apoptotic. Within infected cells, large virus-induced rearrangements of Endoplasmic Reticulum (ER) membranes were visible as well as highly structured virus lattices (Fig 3 Panels B and D). However, at 37°C the CAVA-1 Mahoney infected cells resembled the PBS mock infected samples (Fig 3 Panels A and C). All cells were healthy and not one cell of the >360 cells in the EM preparations showed signs of infection. The input virus was verified by infectious titer determination. The inability to visualize signs of virus replication by EM is in line with the replication kinetics data shown in Fig 1C.

We used intracerebral (i.c.) inoculation in of CD155 transgenic mice [39] to determine whether the temperature sensitive phenotype of the three CAVA vaccine strains translated to *in vivo* attenuation as compared to cIPV strains, and how this level of attenuation compared to that of the Sabin strains (Table 1). Experiment 1 aimed to determine if the CAVA strains were attenuated. For this purpose, three mice per strain were inoculated i.c. at the highest dose (constrained by the maximum inoculation volume and concentration of the virus sample). To confirm results from experiment 1, we inoculated five mice per strain in experiment 2; in parallel the TCID_50_ (infectious units) required to induce paralysis (or death) in 50% of the mice (PLD_50_) was calculated where possible.

**Table 1:** Infectious titers and *in vivo* neurovirulence after intra cerebral inoculation of the CAVA vaccine strains as compared to the cIPV and Sabin strains.

Virus	Virus Titer (log_10_ TCID_50_/ml)	Intra cerebral dose (log_10_ TCID_50_/mouse)	Experiment 1	Experiment 2	PLD_50_ (log_10_ TCID_50_)
CAVA-1 Mahoney	9.9	8.4	0/3*	0/5	>8.4
Sabin 1	9.6	8.0	1/3	2/5	>8.0
Mahoney	10.1	4.0	2/2	5/5	2.0
CAVA-2 MEF-1	9.9	8.4	0/3	0/5	>8.4
Sabin 2	9.2	7.7	0/3	0/5	>7.7
MEF-1	10.0	6.0	2/2	5/5	4.5
CAVA-3 Saukett	9.7	8.2	0/3	0/5	>8.2
Sabin 3	9.9	8.4	0/3	0/5	>8.4
Saukett	9.7	4.0	3/3	5/5	2.6

* Proportion of mice with signs of paresis or paralysis. TCID_50_ = Tissue Culture Infectious Dose 50%, PLD_50_ = Paralytic of lethal dose (50%).

Upon i.c. inoculation of CD155 transgenic mice all three CAVA vaccine strains showed a highly attenuated phenotype. The maximum dose possible (8.2–8.4 Log_10_ TCID_50_/mouse) did not induce paresis or paralysis in any of the mice 21 days post inoculation (Table 1). Similar observations were made for mice inoculated with Sabin strains 2 and 3 when inoculated with the maximum dose possible (7.7 and 8.4 Log_10_ TCID_50_/mouse, respectively). For Sabin 1, a total of three of the eight inoculated mice with the maximum dose of 8.0 Log_10_TCID_50_/mouse showed signs of paresis and/or paralysis. Nonetheless, all three CAVA and Sabin strains were highly attenuated in this model and the PLD_50_ was above the maximum dose tested. By contrast, the neurovirulent cIPV strains induced paralysis in all of the mice at the tested doses (Table 1). The levels of neurovirulence measured here for the cIPV and Sabin strains in this mouse model are in agreement with those reported elsewhere [40,41].

### II. Mechanism of CAVA temperature sensitivity and attenuation

#### Absence of CAVA replication at 37°C is determined at the level of RNA replication and protein translation and requires a combination of mutations in the IRES and Non-Structural proteins

To further investigate the temperature sensitive phenotype associated with the CAVA viruses and potential mechanisms of attenuation, we compared the levels of infectious titers, viral RNA and viral protein translation of the parental Brunenders, CAVA backbone, CAVA-1 Mahoney, and two intermediate viruses containing either the CAVA mutations in the internal ribosomal entry site (IRES) or the CAVA mutations in the Non-Structural proteins in the background of the Brunenders genome (see Fig 2 for a schematic overview of the intermediate viruses).

**Fig 4:**
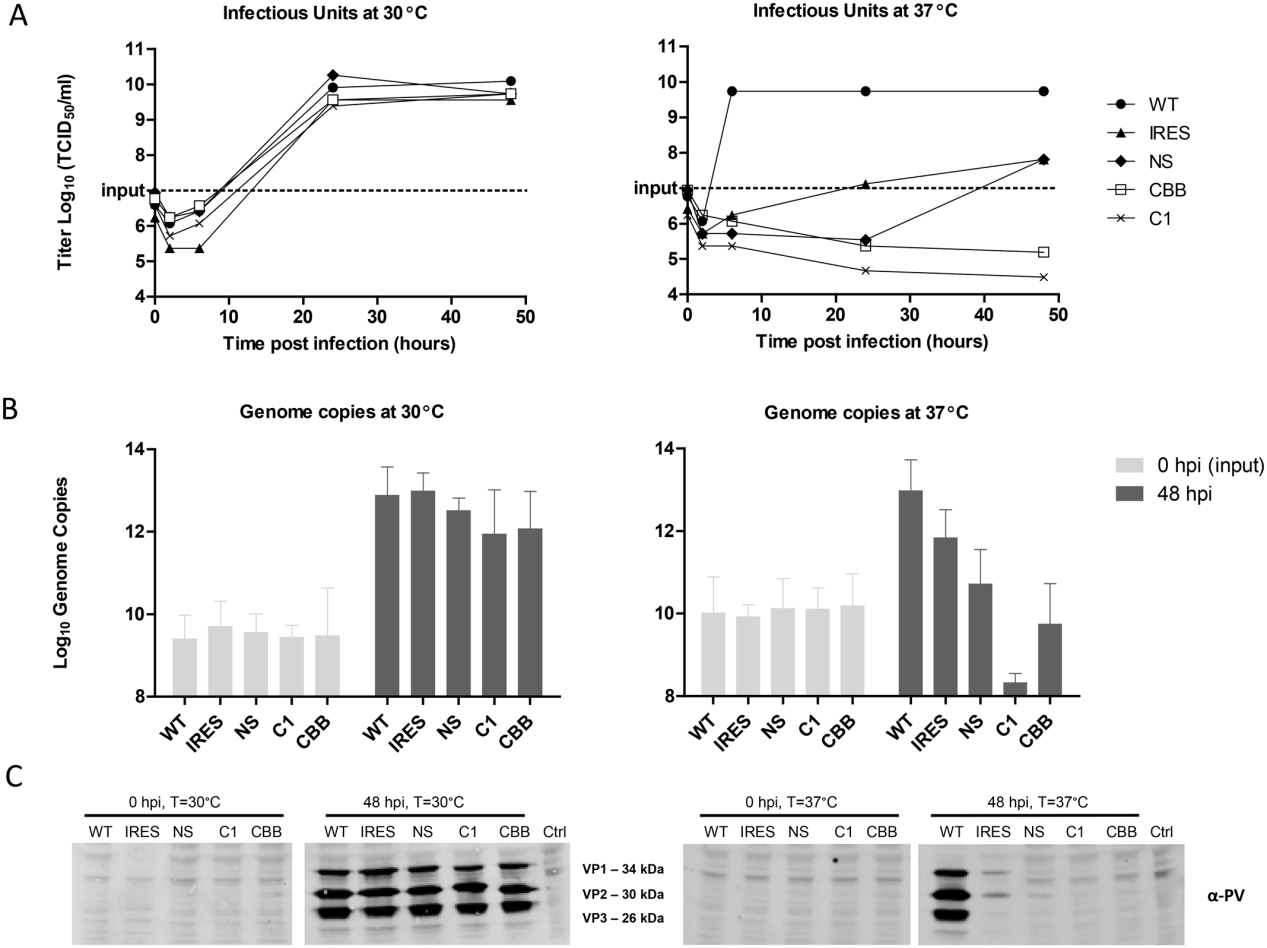
Quantification of poliovirus infectious units (by infectious titer determination, Panel A), viral RNA levels (by RTqPCR, Panel B) and viral proteins (by Western blot, Panel C) after infection of suspension PER.C6 cells with a cell density of 10^7^ cells/ml at an MOI of 1 at 30°C and 37°C, harvested between 0–48 hours post infection. Viruses used were Brunenders (WT), Brunenders with the CAVA mutations in the IRES (IRES), Brunenders with the CAVA mutations in the Non-Structural proteins (NS), the CAVA-1 Mahoney (C1) vaccine strain and the CAVA backbone virus (CBB); Control (Ctrl) is an uninfected control. Data depict one representative infection (n = 1) measured once for infectivity and (n = 3) times for viral RNA and protein levels. Error bars represent standard deviation from the mean and one representative of three independent western blots is shown.

Infections were performed in PER.C6 cells at an MOI of 1, at 30°C and 37°C. Harvests were subjected to infectious titer determination, quantitative reverse transcription PCR (RT-qPCR) and Western Blot analyses. Infection harvests were freeze-thawed and clarified prior to analysis and therefore represent the viral components in the cells and supernatant. At 30°C, all viruses showed similar replication kinetics and maximum infectious titers as compared to the parental Brunenders strain (Fig 4A). At 37°C, CAVA backbone and CAVA-1 Mahoney showed no increase in infectious units (if anything, a decrease) while the intermediate viruses showed a level of temperature sensitivity that was similar to that observed for the three derivative clones from which the CAVA mutations were obtained (Fig 1A); growth kinetics were slower and the maximum titers were approximately 2 logs lower than those obtained by Brunenders.

To study the changes in viral RNA levels during infection, the fold increase of genome copies from 0 to 48 hours post infection were quantified by RT-qPCR (Fig 4B). Viral RNA levels showed average increases of 3.5 and 3.0 log_10_ genome copies for the Brunenders infection at 30°C and 37°C, respectively. By contrast, a decrease in viral RNA levels during infection at 37°C was observed for CAVA-backbone and CAVA-1 Mahoney (0.8 and 1.8 log_10_ decrease in vRNA from 0 to 48 hours post-infection, respectively). For the intermediate viruses, an increase in RNA levels was observed during infection at 37°C that was not to the same extent as observed with the Brunenders parental strain (1.9 and 0.6 log_10_ average increase of genome copies, for viruses containing IRES and Non-Structural mutations, respectively), indicating some impairment for RNA replication for these viruses at 37°C. At 30°C all viruses showed a similar average fold increase in viral RNA levels (2.5–3.3 log_10_ increase in genome copies).

Detection of viral proteins was by performed by Western Blot (Fig 4C) where at the start of infection (0 hours post-infection) no viral proteins could be detected at 37 or 30°C for any of the virus samples. After 48 hours of incubation, similar levels of viral proteins were detectable for all of the viruses at 30°C. At 37°C the Brunenders infection resulted in the clearly visible viral protein bands, whilst infection with the intermediate viruses showed (very) faint viral proteins bands, and infection with the CAVA backbone and CAVA-1 Mahoney viruses did not result in any detectable viral protein bands in the clarified crude harvests. These results indicate that both intermediate viruses are severely impeded in translation, whilst the CAVA-1 Mahoney and CAVA backbone viruses showed no visible signs of viral proteins at all.

#### Identification of specific mutations responsible for the temperature sensitive phenotype

**Fig 5:**
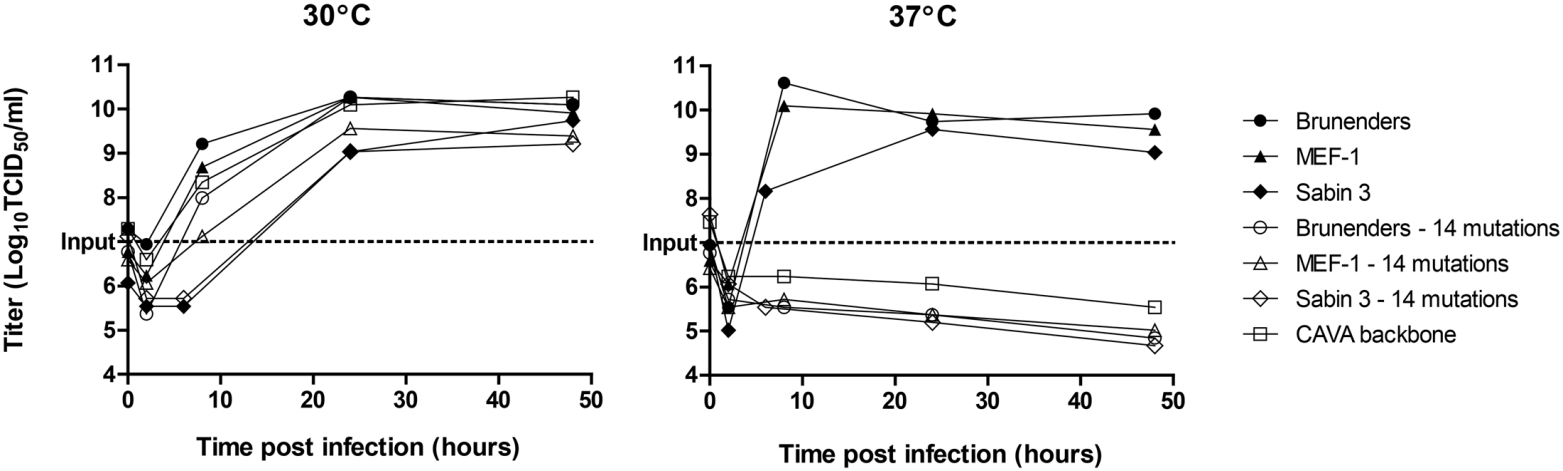
Replication kinetics of poliovirus infection of suspension PER.C6cells with a cell density of 10^7^ cells/ml at an MOI of 1 at 30°C and 37°C harvested between 0–48 hours post infection. Viruses used were Brunenders, Brunenders—with 14 CAVA mutations, MEF-1, MEF-1 –with 14 CAVA mutations, Sabin 3, Sabin 3 –with 14 CAVA mutations and the CAVA backbone virus (see overview of all viruses in Fig 2). The Sabin 3/Sabin 3–14 infection was performed independently from the Brunenders/Brunenders-14 and MEF-1/MEF-1-14 infections. However, the control taken along (CAVA-backbone) was similar for both experiments.

Of the 24 CAVA mutations in the vaccine strains, 14 were selected for synthetic incorporation into different poliovirus backgrounds based on 1) conservation amongst a panel of polioviruses and 2) mutations causing amino acid changes or located in the IRES. The 14 mutations were incorporated in the Brunenders, MEF-1 and Sabin 3 genomes (Brunenders-14, MEF-1-14, and Sabin 3–14, respectively, Fig 2). Incorporation of the 14 CAVA mutations rendered the Brunenders, MEF-1 and Sabin 3 viruses incapable of replication at 37°C (Fig 5). The resulting growth curves were identical to those of the CAVA backbone at 37°C. However, at 30°C all viruses carrying the 14 mutations showed growth curves highly similar to their corresponding parental strains without these mutations. The results show that (a subset of) 14 mutations used here are sufficient to induce the temperature sensitive phenotype.

**Fig 6:**
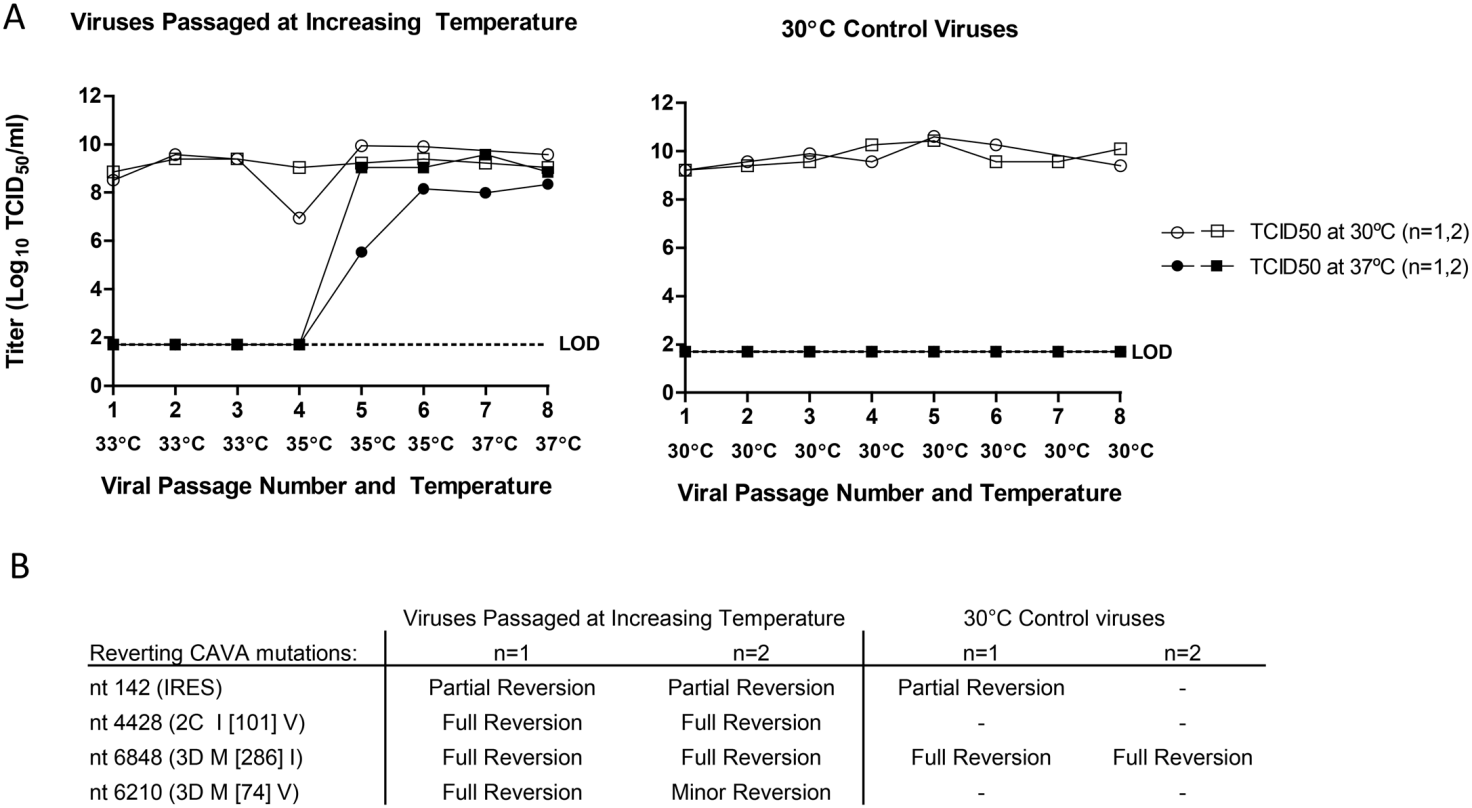
Reversion of the temperature sensitive phenotype by stepwise increase of the infection temperature. Serial passage was performed using CAVA-1 Mahoney in suspension PER.C6 cells infected at a cell density of 10^7^ cells/ml at low MOI (0.01) and harvested at 3–4 days post infection. Temperature was gradually increased (33–37°C) or temperature was kept constant at 30°C for the control viruses. Panel A depicts the viral titers at each passage when titrated and incubated at 30°C or 37°C for the two independent experiments (n = 2). Panel B lists the reverting CAVA mutations of the viruses at passage number 6 where the nucleotide number refers to the position from the start of the viral genome.

To identify more exactly the molecular determinants of temperature sensitivity CAVA-1 Mahoney was serially passaged at 37°C and at low MOI (0.01 TCID_50_/cell), however, this always resulted in an inability to detect quantifiable virus, already after the first passage. Therefore, to select for viruses that had regained the ability to replicate at 37°C, conditions were altered by gradually increasing the infection temperature each subsequent passage. The first three passages were done at 33°C as previous experiments had shown productive infection of PER.C6 cells by the CAVA backbone virus at this temperature (S2 Fig). Temperature was subsequently raised to 35°C for 3 passages and then to 37°C for 2 passages (Fig 6). As a control the same starting virus (CAVA-1 Mahoney) was passaged under the same conditions but at constant temperature (30°C for every passage). The entire passaging experiment was performed twice, independently (n = 2).

During the first four passages the CAVA-1 Mahoney viruses passaged at 30°C and at increasing temperatures retained their temperature sensitive phenotype, as shown by the high virus titers in the crude harvests when titrated and incubated at 30°C, but there was an absence of quantifiable virus when titrated and incubated at 37°C. Capacity to replicate at 37°C was only regained between passage 4 and 5 where infection temperature was gradually increased per passage (Fig 6).

Full viral genome sequencing was performed at passage number 6. Reversion (partial and full) of four CAVA mutations to the nucleotide residues of the parental Brunenders genome was observed in both passaging experiments. Reversions to the Brunenders sequence were observed at nucleotide nt142 in the IRES, in the 2C (nt4428 I [101] V) and 3D (nt6210 M [74] V and nt6848 M [286] I). However, the mutations at nt142 and nt6848 were also observed in viruses that were passaged at 30°C as parallel controls and which still retained the temperature sensitive phenotype, this suggests that these mutations alone do not revert temperature sensitivity. Sequencing of the viruses passaged at increasing temperature also revealed 5 and 6 new mutations across the viral genome, for experiment 1 and 2, respectively. Of these, two mutations (nt127 in the IRES (which forms a base pair with CAVA mutation nt163) and nt918 in VP4 K [58] E) occurred in both experiments.

### III. Characterization of CAVA-1, 2 and 3 as IPV vaccine strains

#### The CAVA vaccine strains are stably attenuated

To evaluate stability of attenuation during vaccine manufacture, the CAVA vaccine strains were tested for *in vivo* neurovirulence after extended *in vitro* passage: five consecutive passages in PER.C6 cells using infection conditions anticipated for vaccine manufacture (temperature = 30°C, MOI = 1, time of harvest = 24 hours post infection), albeit at much smaller scale (V = 10ml). Five passages represent two passages beyond the envisioned commercial manufacturing stage. Passages were performed thrice, independently (n = 3) for each of the three CAVA vaccine strains.

Prior to *in vivo* neurovirulence testing, the *in vitro* temperature sensitive phenotype of all 9 (n = 3 for 3 CAVA strains) passaged viruses was confirmed: growth kinetics at both 37°C and 30°C were identical to the starting CAVA vaccine strains (S3 Fig). Neurovirulence testing of the *in vitro* passaged strains resulted in the same level of *in vivo* attenuation as was observed for the original CAVA vaccine strains (Table 1); the maximum dose possible did not result in symptoms of paresis or paralysis in any of the mice inoculated (n = 3 for experiment 1 and n = 5 for experiment 2). The resultant PLD_50_’s of the passaged vaccine strains were above the maximum doses tested (>8.3, >7.9 and >8.0 Log_10_ TCID_50_ for CAVA-1 Mahoney, CAVA-2 MEF-1 and CAVA-3 Saukett, respectively).

Full genome sequencing of *in vitro* passaged CAVA vaccine strains revealed few mutations after extended passage under production conditions: 1, 0, and 1 mutations for CAVA-1 Mahoney, 0, 0, and 0 mutations for CAVA-2 MEF-1, and 3, 3, and 7 mutations for CAVA-3 Saukett, for each of the three passaging replicates, respectively (S3 Table describes in detail all of the mutations observed after passage). With regard to the 24 inserted CAVA mutations: one of the three CAVA-1 Mahoney passaging replicates showed one partial reversion (nt6848 in the 3D M [286] I). For CAVA-2 MEF-1 no new mutations arose and all 24 CAVA mutations remained present in all of the three (n = 3) passaging experiments. For two of the three CAVA-3 Saukett passaging replicates, the same CAVA mutation (nt142 in the IRES) partially reverted to the parental Brunenders sequence. Nonetheless, the few nucleotide changes observed did not affect the *in vitro* or *in vivo* attenuation (S3 Fig and Table 1). Interestingly, the same CAVA mutations which reverted here (nt142 and 6848) also reverted after 6 passages in the 30°C control passaged viruses (Fig 6) for which no *in vitro* replication at 37°C could be quantified. The frequency of reversion of nt142 and nt6848 suggests that viruses with these mutations encounter a selective disadvantage at ≥30°C.

#### The CAVA vaccine strains are immunogenic *in vivo*

**Fig 7:**
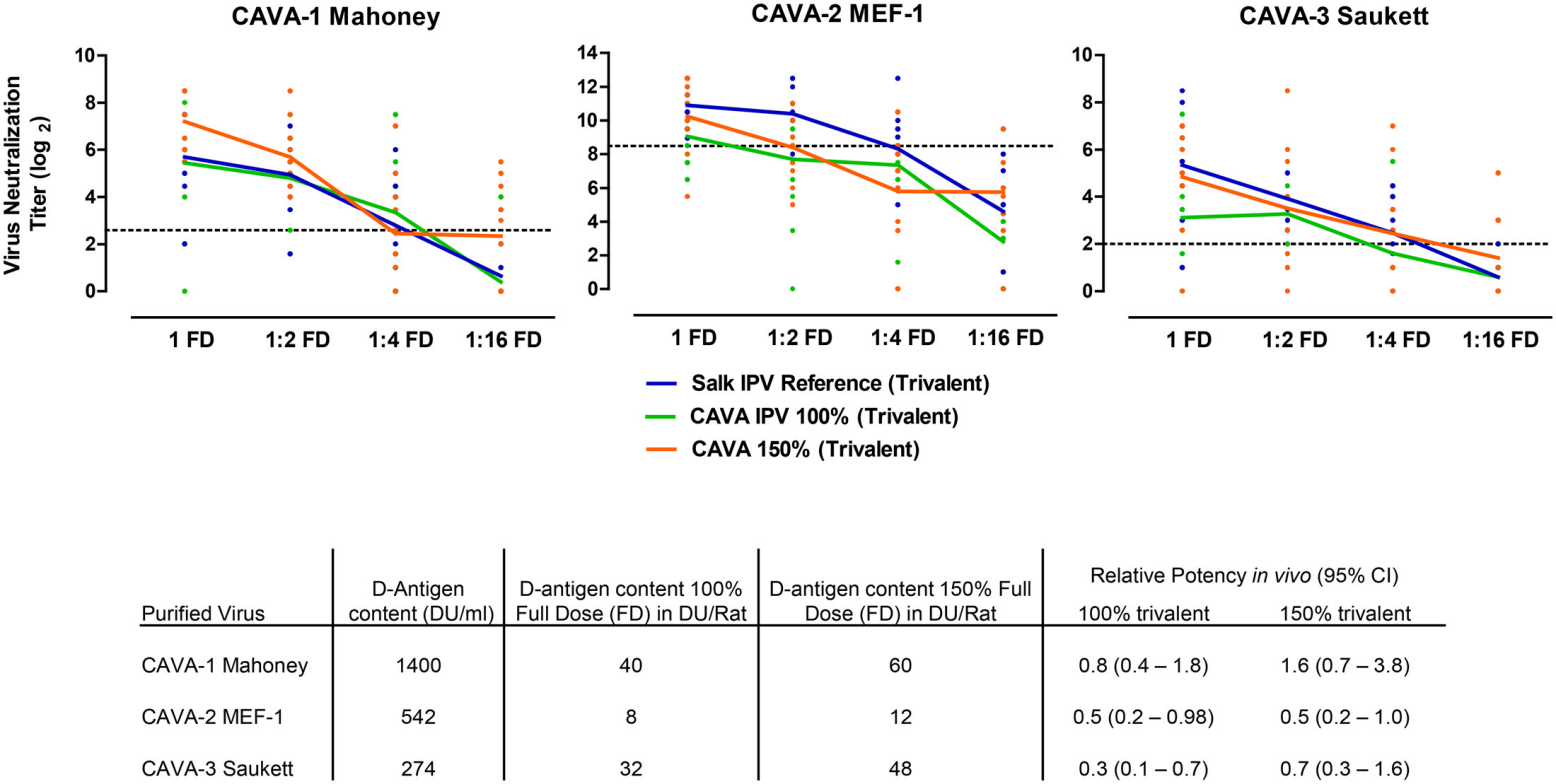
*In vivo* immunogenicity of the CAVA vaccine strains as compared to cIPV. Groups of ten (n = 10) rats were immunized with a full dose (FD: 100% 40:8:32 DU/dose or 150% 60:12:48 DU/dose) or a 1:2, 1:4 or 1:16 dilution of the full dose. Poliovirus type 1, 2 and 3-specific neutralizing antibody titers were determined by Sabin Virus Neutralizing Assay at day 21 post immunization. Each dot represents one individual animal; the connected line represents the geometric mean at each dose. Relative potency estimates and 95% confidence intervals of the difference between the CAVA vaccine strain and cIPV reference based on the number of seroconverting animals are depicted in the table, horizontal dotted line represents the seroconversion limit for each assay.

To determine the immunogenic potential of the CAVA vaccine strains *in vivo*, purified and inactivated material was generated using a scaled down purification and inactivation process which resembles vaccine manufacture. Four groups of 10 rats were immunized with 100% or 150% of a full cIPV dose or a 1:2, 1:4, or 1:16 dilution of the neat dose of the trivalent inactivated CAVA strains. The 100% full dose contained 40, 8, and 32 D-antigen units for serotype 1, 2 and 3, respectively, which is the minimal required dosage for each of the serotypes in cIPV. The 150% full dose contained 60, 12 and 48 D-antigen units for CAVA-1 Mahoney, CAVA-2 MEF-1 and CAVA-3 Saukett, respectively. For each animal, the neutralizing antibody response against Sabin poliovirus was determined three weeks after immunization by Virus Neutralization Assay (VNA). All three CAVA vaccine strains were immunogenic, showing a dose-dependent response and inducing high neutralizing antibody titers in the full dose group (Fig 7) when administered at both 100% and 150% of cIPV dosing. However, the geometric mean neutralization titers induced by CAVA-2 MEF-1 and CAVA-3 Saukett were on average 2 to 3 log_2_ lower in comparison to those induced by the cIPV references when administered at 100% cIPV dose across all dilutions. Relative potency statistical analysis of the 100% dose formulations revealed that CAVA-1 Mahoney was comparable to the cIPV reference; this was not the case for CAVA-2 MEF-1 and CAVA-3 Saukett. Upon increasing the dose to 150% of the cIPV dose the immune response for CAVA-1 Mahoney and CAVA-3 Saukett improved, however, for CAVA-2 MEF-1 this was not as pronounced. Nonetheless, statistical analysis showed that when dosed at 150% of the cIPV dose, all CAVA strains were statistically comparable to the cIPV reference and would have passed a typical batch release test for cIPV (upper 95% confidence limit of relative potency ≥1.0).

## Discussion

As eradication of poliomyelitis draws closer, the poliovirus field is moving towards novel vaccines and vaccination strategies. To serve as novel IPV strains, we generated three attenuated poliovirus strains using a combination of empirical and rational attenuation methods with specific focus on (genetic stability of) attenuation, immunogenicity, and affordability. Our approach for viral attenuation was to develop strains with impaired growth at physiological temperature (≥37°C) with high replicative capacity at (manufacturing) temperature. The CAVA strains were empirically derived by serial passage at low temperature, much like the Sabin strains; however, the subsequent synthetic combination of multiple mutations into one genome was essential to obtain a complete block in viral replication at 37°C.

The CAVA strains showed no sign of successful *in vitro* infection at physiological temperature. Indeed, decreases in infectious units and viral RNA level were measured and no viral proteins or visual signs of infection, such as the presence of replication vesicles or virus lattices, could be observed. This indicates that, at 37°C, the CAVA virus replication cycle is blocked at protein translation, RNA replication, or earlier. Moreover, serial passage at 37°C did not show outgrowth of revertants. Instead all quantifiable virus was lost after the first passage. This suggests that the viruses are locked into the temperature sensitive phenotype by the combination of CAVA mutations, where the inability to replicate also negates reversion and recombination at physiological temperature. To our knowledge, no poliovirus has been described with such a complete block in replication at 37°C. An historic poliovirus strain Mabie (PP3) was reported to lack replicative capacity at 36°C [42], although it induced CPE at physiological temperature denoting maintenance of at least some low level replicative capacity. Impaired growth at physiological temperature has been also extensively shown for more recently described (rationally) attenuated polioviruses [43–48], including the Sabin strains. However, their ability to replicate at 37°C was not abolished, implying a residual risk of reversion and recombination *in vivo*.

The intermediate viruses with CAVA mutations in the IRES or Non-Structural proteins showed impaired, but not completely halted, growth at 37°C. Therefore the combination of CAVA mutations in these regions is required. More specifically, a combination of 14 mutations within those two regions was sufficient to cause the CAVA temperature sensitive phenotype. This was confirmed by introduction of the 14 mutations into other wild-type and attenuated polioviruses of differing serotypes, indicating that the mechanism of action is unique and independent of the parental Brunenders backbone.

**Fig 8:**
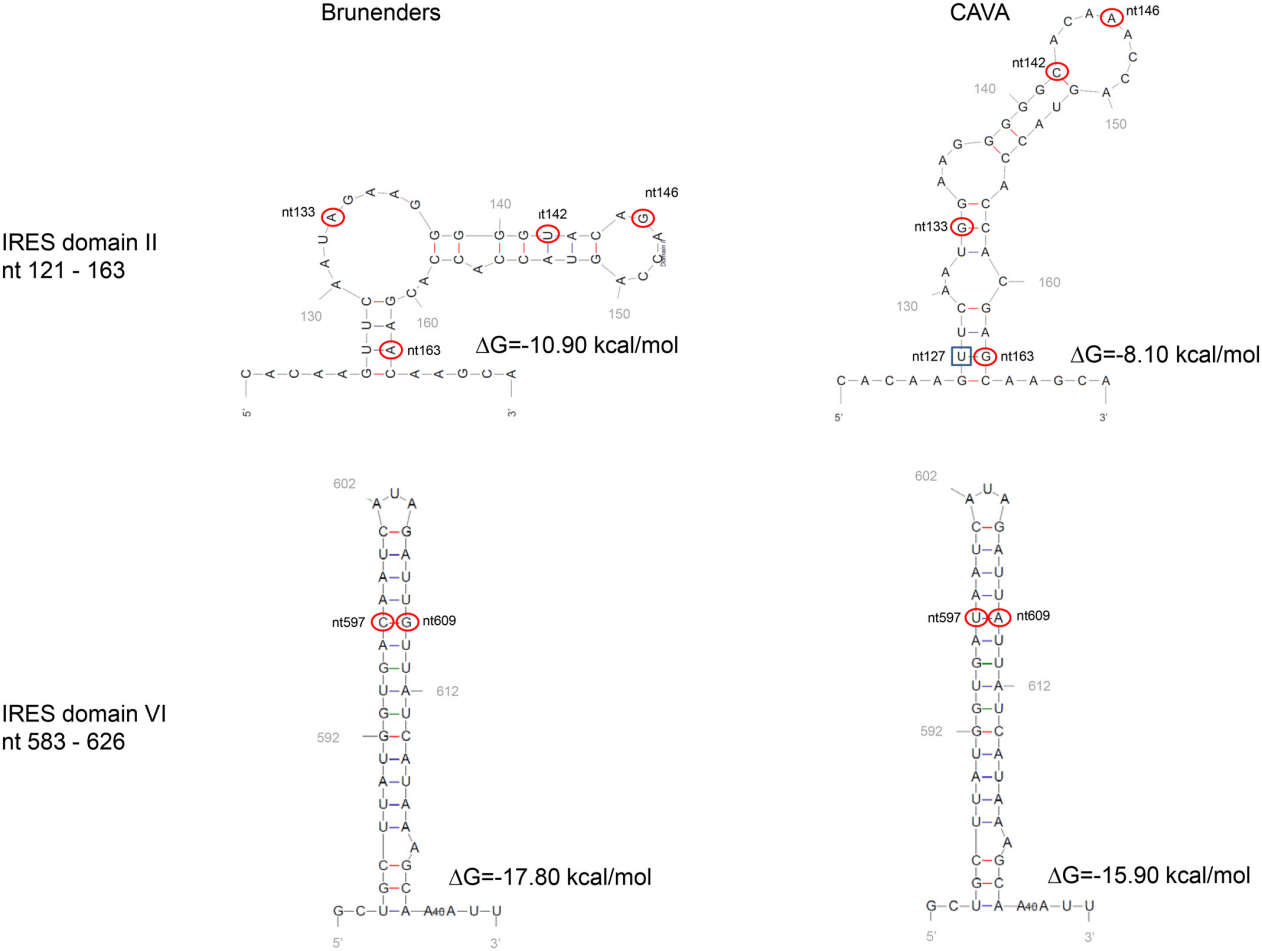
Secondary RNA structure prediction of Domain II and Domain VI of the IRES in Brunenders and CAVA using the MFOLD program developed by M. Zuker. Circled nucleotides (at positions 133, 142, 146, and 163 in domain II and at positions 597, 609 in domain VI) represent nucleotide changes between CAVA and Brunenders. The last remaining CAVA IRES mutation (nt579) lies outside of any IRES domains and in the spacer region between Domains V and VI. After serial passage at increasing temperature (Fig 6) nucleotide 127 (indicated by square box) mutated in both passaging experiments (n = 2) from U to C, forming a C–G base pair with CAVA mutation nt 163. The CAVA mutations induce a change in predicted secondary structure and increased free energy (ΔG) for domain II, whilst for domain VI only the free energy is affected.

The CAVA temperature sensitivity is likely exerted by multiple molecular mechanisms (as exemplified by the synergistic, combinatorial effect of the CAVA mutations) which work together to hamper replication at 37°C. However, at 30°C these mutations do not appear to obstruct virus replication, protein translation, or RNA replication. One explanation for this may be that the introduction of multiple mutations decreases the thermal stability of the viral proteins and/or RNA, resulting in folding defects, conformational changes and subsequent losses of biological functionality of the viral (precursor) proteins and/or functional RNA elements. When environmental thermal energy is lowered (for example at 30°C) the decreased thermal stability may not be sufficient to cause significant changes in protein/RNA structure and function to such an extent that virus replication is restricted. For example, the CAVA mutations in the IRES may destabilize the secondary RNA structure of this essential RNA element. Predicted secondary RNA structures of the CAVA and Brunenders IRES-domains II and VI show that the free energy (ΔG) is raised in the CAVA domains (as well as an altered domain II structure) indicating decreased thermostability (Fig 8). The changes in free energy and structure would likely hamper folding and thermostability of the IRES and therefore disrupt (initiation of) translation. Alterations in Domain II of poliovirus IRESs have previously been reported to show defects in translation [49,50], which gives further credence that these IRES mutations inhibit viral infection at 37°C by hampering translation. However, at lower temperature this intrinsic free energy of the IRES domains may still be permissive for successful protein translation and infection.

The CAVA mutations in the 2C and 3D proteins, which reverted after gradual increase of infection temperature (and which are part of the 14 selected mutations), may play particularly prominent roles in inducing the CAVA temperature sensitivity. However, the compensatory impact of the other CAVA mutations, or the additional new mutations identified after regaining replication capacity at 37°C, cannot be ruled out. The highly conserved 2C protein has multiple functions in host-cell membrane alteration, encapsidation, viral RNA binding and RNA replication [51–54], however, the CAVA mutations in the 2C fall outside any of the known functional domains of this protein. CAVA mutation 4428 at residue 101 is in close proximity to the *cis* acting replication element (cre), an RNA functional domain essential for RNA replication [55]. If the change in nucleotide 4428 alters the secondary structure of the *cre* element in such a way that (at 37°C) replication may be impaired, this may explain part of the temperature sensitive phenotype.

The CAVA mutation 3D[74] is close to residue 73 in the palm of 3D, which in Sabin 1 has been implicated to play a role in temperature sensitivity via a temperature dependent decrease of VPg-uridylation compared to wild-type [56]. It is plausible that the CAVA mutation at residue 74 induces similar temperature sensitive defects in the CAVA viruses.

The CAVA mutation at residue 286 of the 3D is located in the middle finger domain of the polymerase close to a putative translocation domain [57], which is required for nascent RNA chain synthesis. It is conceivable that the amino acid substitution at this position may exert effects during RNA synthesis by temperature dependent, conformational interference with elongation.

Further research is required to pinpoint the exact molecular mechanisms of CAVA temperature sensitivity, and the responsible mutations. However, the vast number of mutations and combinations thereof makes full understanding of the CAVA phenotype a challenging endeavor. To illustrate, even the extensively studied Sabin strains do not have a fully understood molecular mechanism to explain their attenuation [58].

As expected, the inability to propagate at 37°C *in vitro* translated to high attenuation *in vivo*. The CAVA viruses showed a level of attenuation that was at least as high as the Sabin strains, significantly higher than the cIPV strains, and comparable to previously described attenuated poliovirus strains tested in the same animal model [43,45,46]. Follow up studies to this work will focus on comparing the level of attenuation of the CAVA and Sabin strains. This will require the administration of a more sensitive but complicated route of inoculation (i.e. intra spinal). Nonetheless, the intra cerebral model used here is a widely used and accepted model to measure poliovirus neurovirulence which has been used in the field for other attenuated poliovirus strains [41,43,48]. With it the CAVA strains maintained a highly attenuated phenotype after five serial passages, which exceeds the number of passages required to produce final manufacturing seed lots on the PER.C6 cell platform (which likely requires 3 passages). Remarkably, none of the mice inoculated with any of the passaged CAVA strains showed any signs of paralysis. Factors other than passage number, such as infection scale, temperature, cell type, and MOI, can influence the dynamics of a virus population. We attempted to control these variables as far as technically possible, to mimic envisioned vaccine manufacturing conditions. The extended passage performed here showed few alterations in viral sequence and only 0–1 reversions of the 24 incorporated CAVA mutations, with no effects on *in vitro* or *in vivo* attenuation.

Formaldehyde-inactivated versions of the CAVA vaccine strains were immunogenic and induced high neutralizing antibody titers *in vivo* in a standard rat potency assay. CAVA type 2 and 3 components showed reduced vaccine potency relative to the reference cIPV at the same dosing. However, increasing the dose to 150% (of the standard human dose for cIPV) resulted in a significant increase in CAVA immunogenicity, albeit only slightly for type 2, rendering the response of all CAVA strains comparable to the cIPV reference. The reason for the observed lower immunogenicity shown by the CAVA inactivated vaccine is currently unknown. The differences in immunogenicity are most likely induced by the many differences in production processes used to generate the small scale PER.C6-based CAVA IPV and the cIPV international reference standard, which was produced on the Vero cell platform using a validated and optimized process. Virus culture conditions, virus purification, inactivation and/or D-antigen measurements for production of a CAVA-based IPV therefore require further investigation and optimization. Despite the need for further investigation, the CAVA strains showed inactivation rates and D-antigen recoveries during inactivation that were in the same range as those observed for the cIPV strains produced on the PER.C6 cell platform (S4 Fig). Thus the differences observed in immunogenicity are not necessarily induced by differences in formalin inactivation of the CAVA antigens as compared to those of the cIPV strains.

Although unpredicted, another explanation for the slightly lowered immunogenicity could be an incompatibility of the CAVA-backbone with the cIPV capsids and/or the lower culture temperature which may slightly alter the conformation of the virion during virus assembly causing changes in antigenicity and immunogenic potency. Indeed, biophysical characterization of the CAVA vaccine strains as compared to their cIPV counterparts did result in some unexpected differences. For example, antigenicity as measured by D-antigen unit per infectious unit (DU/TCID_50_) was similar for CAVA-1 Mahoney, higher for CAVA-2 MEF-1 and lower for CAVA-3 Saukett as compared to their cIPV counterpart (S4 Table). Furthermore, thermal stability as measured by capsid melting temperature (Tm) showed that all CAVA strains had a melting temperature above 37°C (S5 Table), but that CAVA-2 MEF-1 and CAVA-3 Saukett displayed significantly lower Tm as compared to their cIPV counterpart. The biological relevance of these *in vitro* differences and their impact on the ultimate vaccine immunogenicity in rats is currently unknown and therefore requires further investigation. Currently, work is in progress to determine whether the observed differences in immunogenicity are intrinsic to the CAVA viruses or caused by the differing production processes of the CAVA vaccine and the cIPV reference standard. It is important to strive for comparability to cIPV immunogenicity as this vaccine has been used successfully for more than half a century inducing high titers of antibodies with an extensive duration of protection against poliomyelitis. A similar immunogenic profile may therefore facilitate clinical development of a novel vaccine candidate.

Cost of IPV is a critical parameter to enable immunization of the developing world, an essential endeavor to achieve and maintain eradication. Therefore, strategies to increase IPV affordability are encouraged by the WHO[59]. Virus production during vaccine manufacture with high yields can significantly decrease costs of goods. We have previously demonstrated that the use of the PER.C6 platform increases volumetric productivity of infection harvests as compared to the Vero-based platform for cIPV strains [60] and for the Sabin strains [61]. Use of the highly productive PER.C6 platform for propagation of the CAVA vaccine strains resulted high infectious titer yields (9.4–9.9 Log_10_ TCID_50_/ml), comparable to those reached with the Sabin strains (9.2–9.9 Log_10_ TCID_50_/ml), which demonstrates potential for significantly reducing cost of goods and consequent vaccine pricing required for global roll-out of an affordable IPV.

Next generation IPV vaccine strains should ideally portray a non-infectious phenotype to reduce the risk of transmission and disease, should dissemination into the environment occur. The novel CAVA strains are characterized by an inability to replicate at 37°C and capacity to propagate to high titers at 30°C. Their unprecedented temperature sensitivity translated to a high level of *in vivo* neuroattenuation and suggests that the CAVA strains are non-infectious at physiological temperature. Their use can therefore decrease biosafety risks associated with cIPV manufacturing. These novel attenuated strains are designed to be antigenically equal to the cIPV strains, although further work is required to demonstrate equivalent immunogenicity. In combination with the highly productive PER.C6 cell culture platform, the stably attenuated CAVA strains may serve as an attractive low-cost and (bio)safe option for the production of a next generation IPV which can aid in achieving and maintaining a polio free world.

## Materials and Methods

### Cells, virus rescue and infections

The Brunenders, MEF-1 and Saukett viruses were derived from virus seeds kindly donated by SBL (former Swedish Bacteriological Laboratories). Sabin 1, 2 and 3 were purchased at The National Institute for Biological Standards and Control (NIBSC, catalogue number: 01/528, 01/530, and 01/532, respectively). The Mahoney virus was purchased at the European Virus Archive (EVA).

All remaining viruses used were rescued via RNA transfection for which the RNA was transcribed *in vitro* with a T7 polymerase using a synthetic cDNA plasmid as a template, as described previously [38,62]. The cDNA plasmids were synthetically generated at Genscript and contained the entire viral genome sequence downstream of a T7 promoter. A schematic overview of all synthetic viruses and the incorporated mutations is shown in Fig 2.

PER.C6 cells [63] (Janssen proprietary cell line, derived from primary human retina cells) were maintained as described previously [60]. All infections were performed in suspension PER.C6 cells using a cell seeding density of 10^7^ viable cells per ml and infection volumes ranged between 5–15 ml in shaker flasks to 250 ml in roller bottles. Infections were performed at differing temperatures (26–30, 33, 35 or 37°C) as well as MOI (0.01–2 TCID_50_/cell), indicated per experiment. Time of Harvest ranged from t = 0–4 days post infection. Brunenders was passaged 34 times at low MOI (0.01 TCID_50_/cell) on PER.C6 cells with 10^7^ cells/ml at 26–30°C.

### Assays

Infectious titer determination was performed in multi-well 96 plates seeded with 6.5x10^4^ adherent PER.C6 cells per well in DMEM supplemented with 10% FBS and 10mM Magnesium Chloride. Eleven serial virus dilutions with a five-fold dilution factor were prepared and added to the cells with subsequent incubation for 13 days at 30°C for all titration assays, unless indicated differently. On day 13 each well was scored for CPE and titers were calculated by method of Spearman and Kärber [64].

EM was performed at Leiden University Medical Center. Infection harvests were fixed at 1 hour at room temperature in 1.5% glutaraldehyde in 0.1 M cacodylate buffer (pH 7.4) and stained with 1% osmium tetroxide for 1 hour. Samples were pelleted in 3% agar where resulting pellets were cut and gradually dehydrated with an ethanol series. The samples were then infiltrated for 1 hour with a 1:1 mixture of propylene oxide and epoxy LX-112 resin (Ladd Research). After an additional hour in 100% epoxy LX-112, the samples were polymerized for 48 h at 60°C. Cell sections of 50 nm were cut, placed onto carbon-coated formvar grids, and counterstained with 7% uranyl acetate and lead citrate for 20 and 10 minutes, respectively. Imaging was performed with a Tecnai 12 BioTwin transmission electron microscope (FEI company) operated at 120 kV.

For detection of viral proteins 100 μg of total protein from clarified crude harvests (after 3 freeze thaw cycles) was loaded into a NuPAGE Novex Bis-Tris 4–12% protein gel (Life Technologies) and blotted onto Nitrocellulose membranes (Life Technologies). Membranes were blocked in 5% non-fat dried milk (Bio-Rad) and incubated overnight with a 1:1000 dilution of goat polyclonal antibodies against poliovirus types 1,2,3 (ProSci), followed by 2 hours with a 1:15000 dilution of donkey anti-goat IRDye 800CW secondary antibody (Westburg). Proteins were visualized using Odyssey infrared imaging system (Li-Cor, BioSciences).

Quantification of poliovirus RNA was performed by RT-qPCR using viral RNA isolated from clarified, freeze-thawed infection harvests using a QIAamp viral RNA isolation kit (Qiagen). Viral RNA was reverse transcribed to cDNA and subsequently amplified with the Power SYBR Green RNA-to-Ct 1-Step Kit (Life Technologies), using 400nM forward primer (5’ TCTCCTAGCCCAATCAGGAA 3’) and 400nM reverse primer (5’ TCTCCCATGTGACTGTTTCAA 3’) flanking an amplicon (86nt in length) in the 3D polymerase gene. Real-time PCR was performed in a 7500 Fast thermocycler (Life Technologies) starting with 30 min at 48°C for reverse transcription and 10 min at 95°C for activation of DNA polymerase, followed by 40 amplification cycles of 15 sec at 95°C for denaturation and 1 min at 63°C for annealing and extension. Purified, *in vitro* transcribed RNA of the CAVA backbone virus, with known concentration number of poliovirus genome copies, was used as a standard to allow RNA quantification of tested samples.

Sequencing of the full viral genomes was performed by RT-PCR and Sanger sequencing as described previously [38].

RNA secondary structures predictions of the IRES domains II and IV of CAVA and Brunenders were executed by the MFOLD program (http://mfold.rna.albany.edu/?q=mfold/RNA-Folding-Form) developed by M. Zuker.

### *In vivo* attenuation in transgenic mice

Neurovirulence testing was performed at Stony Brook University using transgenic mice expressing the poliovirus receptor (CD155) [39]. For each poliovirus serotype studied, they were 3 experimental groups (CAVA and Sabin strains) and one control group (wild-type strains). Two to three mice were housed per cage. For the neurovirulence testing, groups of three and five (n = 3 and n = 5) CD155 transgenic mice (8±2 weeks of age, male and female) were anesthetized and then inoculated i.c. (30 μL per mouse, dose in TCID_50_ per mouse, Table 1). For the wild-type cIPV strains (control group) doses ranged from 10^2^–10^6^ TCID_50_ per mouse. All inoculations took placed in a biosafety cabinet type II. Intraperitoneal injection of Ketamine (100 mg/kg)/ Xylazine (10 mg/kg) combination was used as anesthetic producing short-term surgical anesthesia with good analgesia facilitating i.c inoculation in the anesthetized mice. After inoculation, mice were examined daily for 21 days for signs of paresis or paralysis, where scoring was done according to the WHO’s Standard Operating Procedure for poliovirus neurovirulence testing in transgenic mice [65]. The virus titer that induced paralysis (or death) in 50% of the mice ((P)LD_50_) was calculated by the method of Reed and Muench [66].

### Virus purification and inactivation

CAVA harvests were treated with domiphen bromide to remove host cell DNA and consequently clarified of a series of filters. Prior to Cation Exchange Chromatography (CEX) the clarified harvests were acidified using 25mM sodium citrate. CEX was performed using Sartobind S cationic membranes. The CEX eluate was subjected to a Size Exclusion Chromatography step for further purification (polish) and buffer exchange. The SEC eluate was conditioned using M199 and glycine prior to inactivation. Inactivation was performed according to the EP guidelines and in line with Salk’s description of poliovirus inactivation procedure in the 1950s [67]. Briefly, the purified batches were filtered (0.22μm) prior to formalin addition (0.009% formalin or 3.3mM formaldehyde) and incubated for 13 days at 37°C and shaking at 75 rpm. Filtration was performed at days 6 and 13 of inactivation. The inactivated purified virus bulks were tested for purity (OD and SDS PAGE) and sterility (mycoplasma, endotoxin, bioburden).

### *In vivo* immunogenicity in rats

Rat Potency testing was performed at the National Institute of Biological Standardization (NIBSC).

The purified and inactivated monovalent CAVA samples were tested for D-antigen content by ELISA. This D-antigen ELISA utilizes polyclonal capture and monoclonal detection antibodies raised against active Sabin viruses. The inactivated CAVA viruses were consequently tested for monovalent *in vivo* immunogenicity in the rat potency model. Four groups of Wistar female rats (n = 10) were immunized with a full dose, or a 1:2, 1:4 and 1:16 dilution of the full dose of each of the inactivated CAVA vaccine strains, or the reference vaccine BRP2. The 100% full human dose represents 40, 8 or 32 D-antigen units of Type 1, 2 and 3 respectively, which is the minimal required dosing of cIPV. The 150% full human dose represents 60, 12 or 48 D-antigen units of Type 1, 2 and 3 respectively. After three weeks, sera were collected. Neutralizing antibodies against all three poliovirus types were measured in separate assays using 100 TCID_50_ of Sabin 1, Sabin 2 or Sabin 3 poliovirus strains as assay challenge viruses and Hep2C as indicator cells. Sera-virus incubation was overnight at 4°C, followed by 3 hours at 35°C [68]. Assay was stopped after 6 days of incubation at 35°C by staining the plates with Naphthalene black. Virus neutralization titers were expressed as a score based on the last serum dilution with no signs of cytopathic effect (CPE). Relative Potency was calculated based on the number of seroconverting animals for each vaccine in relation to the reference BRP2 using Combistats analysis software. This was performed for each poliovirus serotype separately. Current NIBSC seroconversion limits are ≥4, ≥362, and ≥6 for types 1, 2 and 3, respectively, and are set based on a minimum of three repeated tests with the reference vaccine. A cut-off value is determined as the mid-point on a log2 scale of the minimum and maximum geo-mean titers, according to the European Pharmacopoeia [68].

### Ethics statement

All mice used for *in vivo* neurovirulence testing at Stony Brook University have been maintained under specific-pathogen-free conditions and animals experiments were performed in strict compliance with the national guidelines provided by “The Guide for Care and Use of Laboratory Animals” and The Stony Brook University Institutional Animal Care and Use Committee (IACUC). The IACUC of the Stony Brook University approved all animal experiments presented here (permit #267166). CD155 transgenic mice were bred in the Division of Laboratory Animal Resources (DLAR) at Stony Brook University. All mice were housed in a pathogen-free mouse facility at the DLAR facility.

NIBSC’s Animal Welfare and Ethical Review Body approved the application for Procedure Project Licence Number 80/2523 which was approved by the UK Government Home Office and under which animal care and protocols for Rat potency testing were conducted. All animal care and protocols used at NIBSC adhere to UK regulations (Animals, scientific procedures, Act 1986 that regulates the use of animals for research in the UK) and to European Regulations (Directive 2010/63/EU of the European Parliament on the protection of animals used for scientific purposes). The experiments in rats shown here were carried out following protocol 1 within Home Office Procedure Project Licence Number 80/2523 referred above.

## Supporting Information

S1 Fig Replication kinetics of Brunenders virus after 11 and 28 passages in PER.C6 cells at low MOI (0.01) and low temperature (≤30°C). Viruses were capable of replication at 37°C.
(PPT)

S2 Fig Replication kinetics of CAVA backbone virus at 33°C in PER.C6 cells. (PPT)

S3 Fig Replication kinetics of CAVA vaccine strains after 5 *in vitro* passages at 30°C in PER.C6 cells (n = 3) as compared to starting CAVA vaccine strain. (PPT)

S4 Fig Inactivation rates and D-antigen recoveries after formaldehyde inactivation of the CAVA, Sabin and cIPV strains. (PPT)

S1 Table Detailed description of all 31 CAVA mutations derived from the three independent clones. (DOCX)

S2 Table *In vivo* neurovirulence of the CAVA backbone in transgenic CD155 mice (PLD_50_) after intra cerebral, intra peritoneal and intra muscular administration. (PPT)

S3 Table Detailed description of the mutations observed as compared to the starting stock after genetic stability passaging of the three CAVA Vaccine strains for the three independent experiments (n = 3). (DOCX)

S4 Table Infectious titer, D-antigen content (quantified by D-antigen ELISA as performed in [60] and ratio of D-antigen units per infectious unit (DU/TCID_50_) of CAVA viruses after PER.C6 cell infection at 30°C and cIPV strains after PER.C6 cell infection at 35°C. An unpaired t-test was performed to assess if the difference in DU/TCID_50_ ratio between the CAVA strains and the respective cIPV strain is significant (two-tailed, α = 0.05). P-values are shown for each combination and an asterisk (*) represents a significant difference.
(DOCX)

S5 Table Capsid melting temperatures (Tm) of the CAVA viruses and their cIPV counterparts. Protocol adapted from [69] briefly, crude harvests were clarified over a series of filters (Clarified virus samples) and subsequently purified by Cation exchange and Size exclusion chromatography (Purified virus samples). Forty μl of Quantifluor dye (2000x diluted in double distilled H2O; Promega) was added to all sample solutions and three replicates per sample were transferred to a 96-wells PCR plate at 50 μl/well. The plate was covered with an optical adhesive film and placed in the 7500 Fast Real-Time PCR System (Applied Biosystems). The machine was set to ramp from 30°C to 79°C, taking a fluorescence reading at every 0.5°C increase per 30 seconds. Temperature melting point (Tm) was determined by calculation of the inflection point (the intercept of the second derivative of the x-axis) of the raw fluorescence data. An unpaired t-test was performed to assess if the difference in Tm between the CAVA strains and the respective cIPV strain is significant (two-tailed, α = 0.05). P-values are shown for each combination and an asterisk (*) represents a significant difference.
(DOCX)
