# Supplementary figures and images for: Cold-Adapted Viral Attenuation (CAVA): Highly Temperature Sensitive Polioviruses as Novel Vaccine Strains for a Next Generation Inactivated Poliovirus Vaccine
Source: PLoS Pathog. 2016 Mar 31;12(3):e1005483. doi: 10.1371/journal.ppat.1005483 (PMC4816566; doi:10.1371/journal.ppat.1005483)

## Slide 1
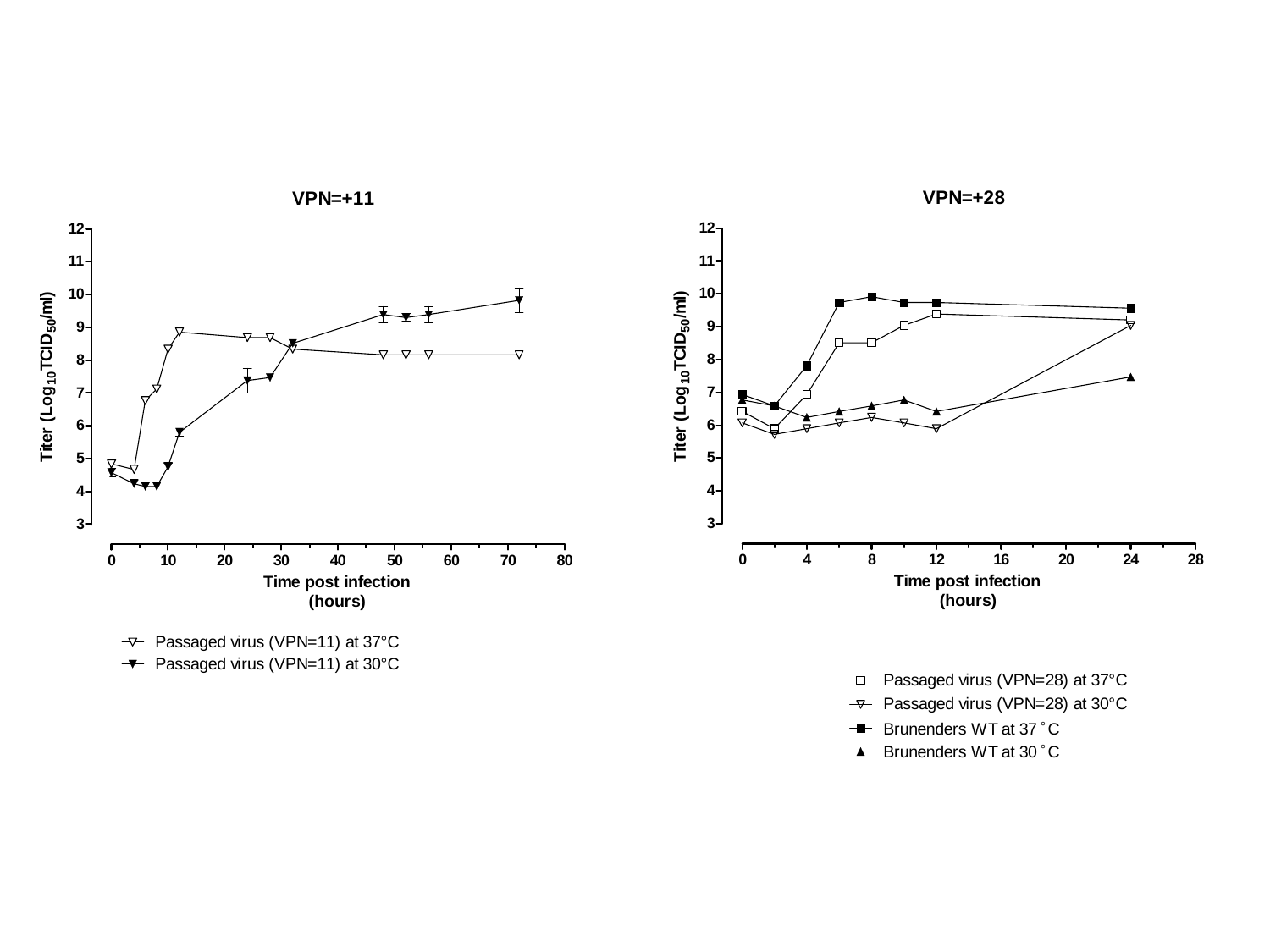

Supplement: S1 Fig — Viruses were capable of replication at 37°C. (PPT) [file ppat.1005483.s001.ppt]

## Slide 1
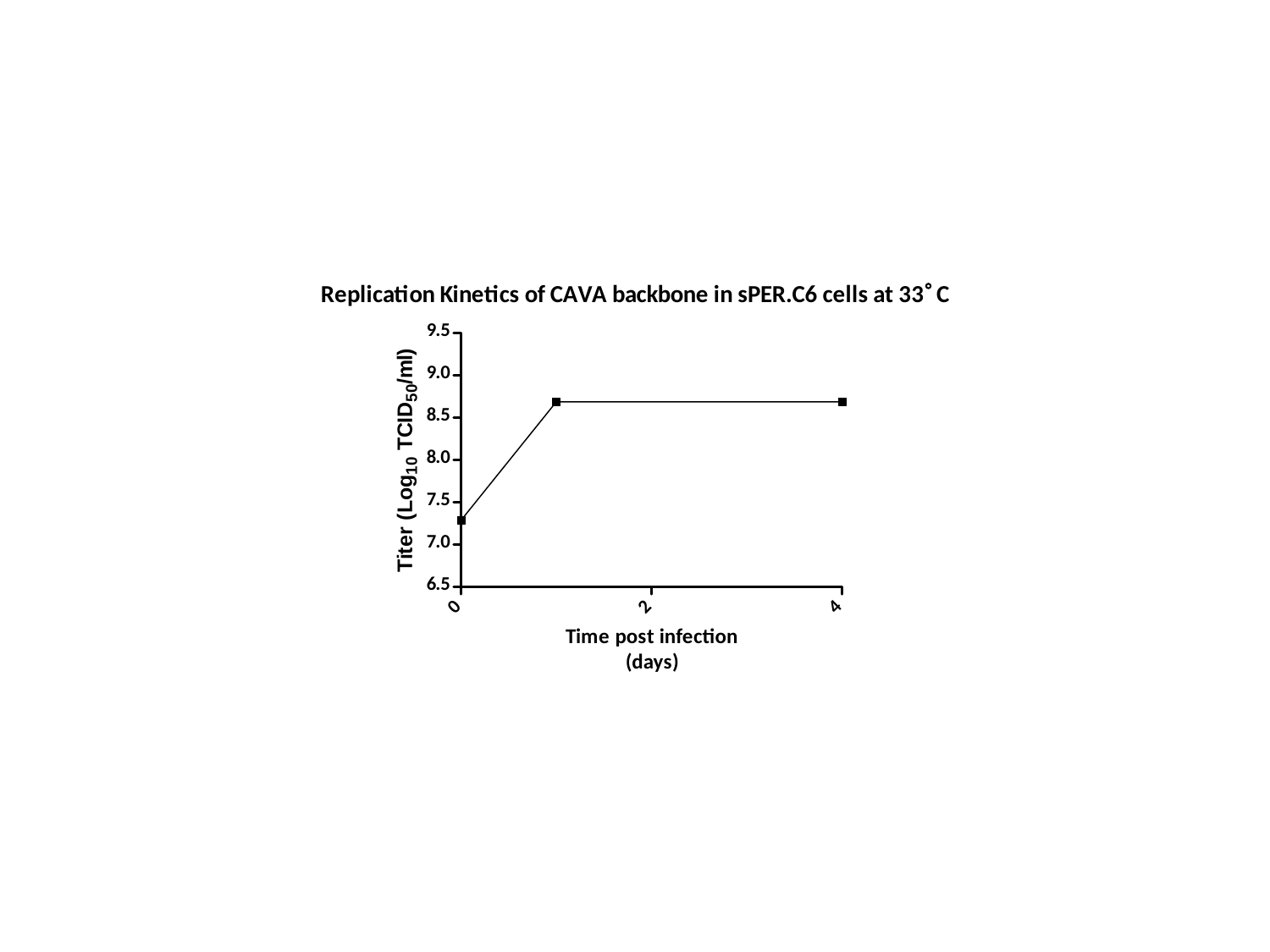

Supplement: S2 Fig — (PPT) [file ppat.1005483.s002.ppt]

## Slide 1
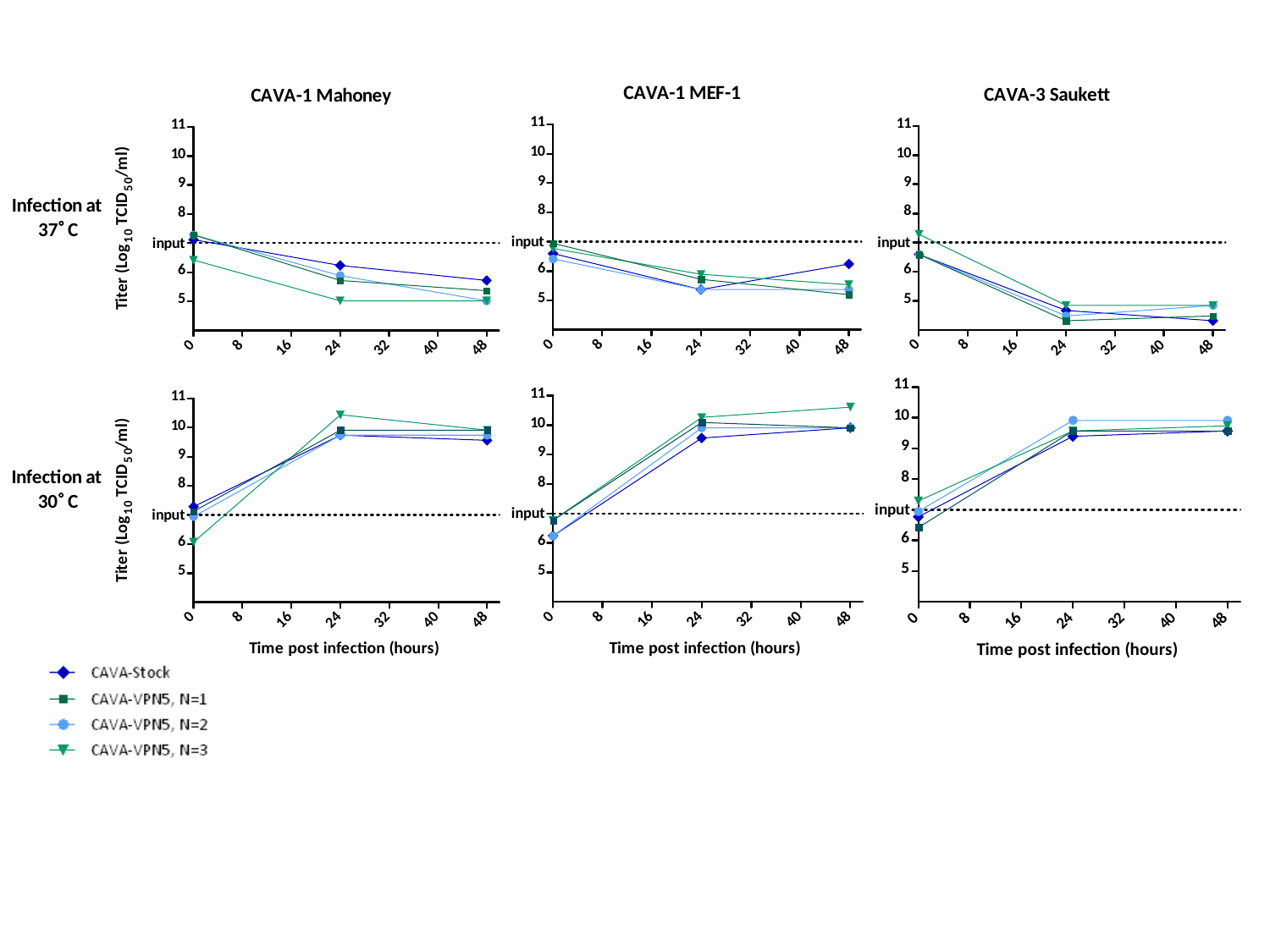

Supplement: S3 Fig — (PPT) [file ppat.1005483.s003.ppt]
