# Supplementary material for: Cold-Adapted Viral Attenuation (CAVA): Highly Temperature Sensitive Polioviruses as Novel Vaccine Strains for a Next Generation Inactivated Poliovirus Vaccine
Source: PLoS Pathog. 2016 Mar 31;12(3):e1005483. doi: 10.1371/journal.ppat.1005483 (PMC4816566; doi:10.1371/journal.ppat.1005483)
Supplement: S4 Fig — (PPT) [file ppat.1005483.s004.ppt]

## Slide 1
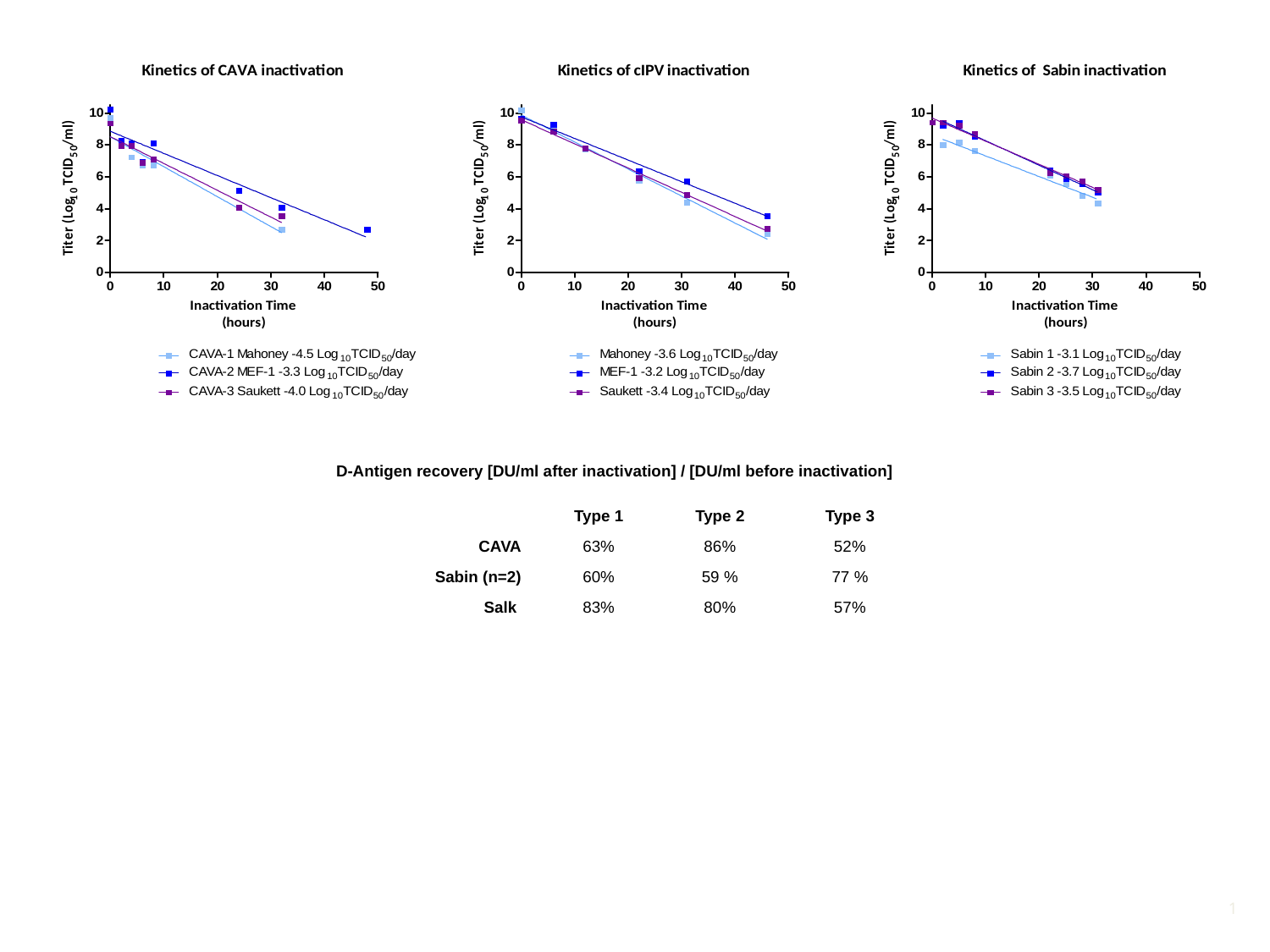

| D-Antigen recovery [DU/ml after inactivation] / [DU/ml before inactivation] | | | |
| --- | --- | --- | --- |
| | Type 1 | Type 2 | Type 3 |
| CAVA | 63% | 86% | 52% |
| Sabin (n=2) | 60% | 59 % | 77 % |
| Salk | 83% | 80% | 57% |
<number>
