# Supplementary material for: Cold-Adapted Viral Attenuation (CAVA): Highly Temperature Sensitive Polioviruses as Novel Vaccine Strains for a Next Generation Inactivated Poliovirus Vaccine
Source: PLoS Pathog. 2016 Mar 31;12(3):e1005483. doi: 10.1371/journal.ppat.1005483 (PMC4816566; doi:10.1371/journal.ppat.1005483)
Supplement: S1 Table — (DOCX) [file ppat.1005483.s005.docx]

| Region | nt# | nt change | aa change |
| --- | --- | --- | --- |
| 5'UTR | **133^A,B^** | A → G | - |
|  | **142^B^** | T → C | - |
|  | 146^A,B^ | G → A | - |
|  | **163^B^** | A → G | - |
|  | 579^C^ | G → A | - |
|  | **597^A,B^** | C → T | - |
|  | **609^C^** | G → A | - |
| VP4 | 805^A,B,C^ | A → C | Y [20] S |
| VP3 | 1787^A, B,C^ | C → T | - |
|  | 1905^C^ | T → C | - |
| VP1 | 2756^A^ | T → C | - |
|  | 3236^A,B^ | C → T | - |
|  | 3323^C^ | C → T | - |
|  | 3376^A,B,C^ | A → G | E [298] G |
| 2A | 3476^C^ | C → T | - |
|  | **3486^A,B,C^** | G → A | V [33] I |
| 2B | **3852^A,B^** | A → T | I [6] F |
|  | **4120^A,B,C^** | T → C | I [95] T |
| 2C | 4253^A,B^ | C → T | - |
|  | 4301^C^ | T → C | - |
|  | **4428^A,B^** | A → G | I [101] V |
|  | **4563^A,B,C^** | A → T | I [146] L |
|  | 4811^C^ | A → G | - |
| 3B | **5436^C^** | G → A | V [21] M |
| 3C | 5705^C^ | A → G | - |
| 3D | 6059^A,B,C^ | C → T | - |
|  | **6210^A^** | A → G | M [74] V |
|  | 6488^B^ | C → T | - |
|  | **6848^C^** | G → A | M [286] I |
|  | 7079^A^ | T → C | - |
|  | **7102^A,B,C^** | T → C | V [371] A |

Nucleotide numbering refers to the start the viral genome. The bold and underlined mutations represent the 14 mutations incorporated into the Brunenders – CAVA 14 mutations, MEF-1 CAVA 14 mutations and Sabin 3 – 14 CAVA mutations viruses (see Fig 5). ^A^ mutations derived from Clone A, ^B^ mutations derived from Clone B, ^C^ mutations derived from Clone C. nt= nucleotide, aa=amino acid
