# Supplementary material for: Cold-Adapted Viral Attenuation (CAVA): Highly Temperature Sensitive Polioviruses as Novel Vaccine Strains for a Next Generation Inactivated Poliovirus Vaccine
Source: PLoS Pathog. 2016 Mar 31;12(3):e1005483. doi: 10.1371/journal.ppat.1005483 (PMC4816566; doi:10.1371/journal.ppat.1005483)
Supplement: S2 Table — (PPT) [file ppat.1005483.s006.ppt]

## Slide 1
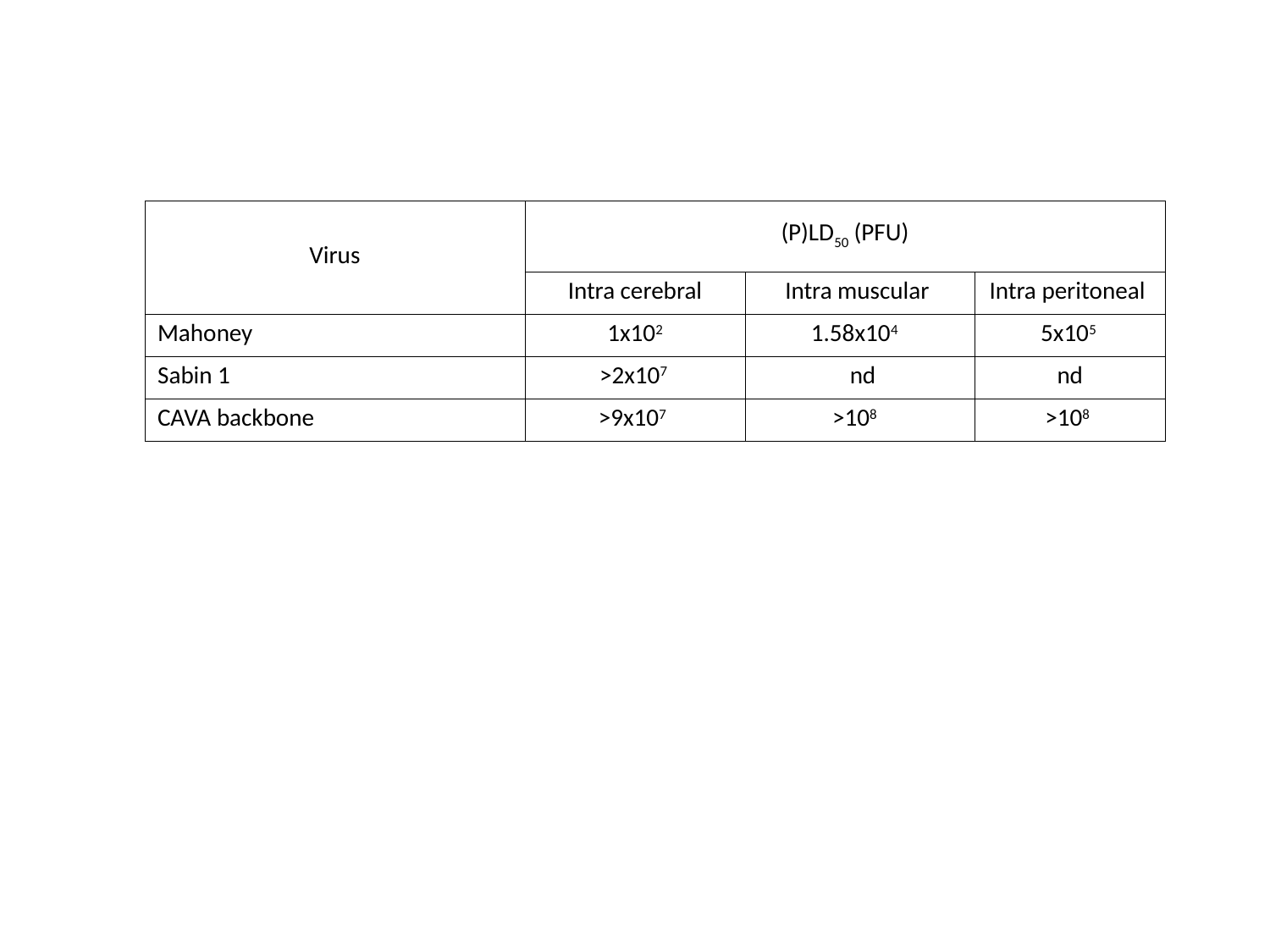

| Virus | (P)LD50 (PFU) | | |
| --- | --- | --- | --- |
| | Intra cerebral | Intra muscular | Intra peritoneal |
| Mahoney | 1x10^2^ | 1.58x10^4^ | 5x10^5^ |
| Sabin 1 | >2x10^7^ | nd | nd |
| CAVA backbone | >9x10^7^ | >108 | >108 |
