# Supplementary material for: Cold-Adapted Viral Attenuation (CAVA): Highly Temperature Sensitive Polioviruses as Novel Vaccine Strains for a Next Generation Inactivated Poliovirus Vaccine
Source: PLoS Pathog. 2016 Mar 31;12(3):e1005483. doi: 10.1371/journal.ppat.1005483 (PMC4816566; doi:10.1371/journal.ppat.1005483)
Supplement: S3 Table — (DOCX) [file ppat.1005483.s007.docx]

| Virus | Number of mutations | Detailed description of mutations from virus after extended *in vitro* passage |
| --- | --- | --- |
| CAVA-1 Mahoney VPN=+5 (n=1) | 1 | VP4 K[58]E |
| CAVA-1 Mahoney VPN=+5 (n=2) | 0 | None |
| CAVA-1 Mahoney VPN=+5 (n=3) | 1 | 3D M[286]I* |
| CAVA-2 MEF-1 VPN=+5 (n=1) | 0 | None |
| CAVA-2 MEF-1 VPN=+5 (n=2) | 0 | None |
| CAVA-2 MEF-1 VPN=+5 (n=3) | 0 | None |
| CAVA-3 Saukett VPN=+5 (n=1) | 3 | VP2 A[165]G**, VP1 K[37]N, VP1 L[227]J |
| CAVA-3 Saukett VPN=+5 (n=2) | 3 | nt142 IRES*, VP3 E[39]K, VP1 V[64](silent) |
| CAVA-3 Saukett VPN=+5 (n=3) | 7 | nt142 IRES* , nt137 IRES, VP2L[101]I, VP3 E[39]K, VP3 P[142]H, VP3N[205], VPN1L[227]I |

Nucleotide numbering refers to the start the viral genome. *Partial CAVA reversion to Brunenders, **Change in Antigenic site 2B.
