# Supplementary material for: Cold-Adapted Viral Attenuation (CAVA): Highly Temperature Sensitive Polioviruses as Novel Vaccine Strains for a Next Generation Inactivated Poliovirus Vaccine
Source: PLoS Pathog. 2016 Mar 31;12(3):e1005483. doi: 10.1371/journal.ppat.1005483 (PMC4816566; doi:10.1371/journal.ppat.1005483)
Supplement: S4 Table — An unpaired t-test was performed to assess if the difference in DU/TCID50 ratio between the CAVA strains and the respective cIPV strain is significant (two-tailed, α = 0.05). P-values are shown for each combination and an asterisk (*) represents a significant difference. (DOCX) [file ppat.1005483.s008.docx]

| **Virus Sample** | **D-antigen content (DU/ml)** | **Titer (Log_10_TCID_50_/ml)** | **DU/TCID_50_ (x10^-7^)** | |
| --- | --- | --- | --- | --- |
| CAVA-1 Mahoney Average (N=5) ± SD | 2071 ± 368 | 9,67 ± 0,32 | 5,3 | p = 0.1951 |
| Mahoney Average (N=3) ± SD | 3366 ± 436 | 10,17 ± 0,01 | 2,3 |  |
| CAVA-2 MEF-1 Average (N=5) ± SD | 707 ± 164 | 9,63 ± 0,27 | 1,7 | p =0.048* |
| MEF-1 Average (N=3) ± SD | 518 ± 40,7 | 10,04 ± 0,06 | 0,5 |  |
| CAVA-3 Saukett Average (N=5) ± SD | 312 ± 116 | 9,70 ± 0,34 | 0,8 | p = 0.001* |
| Saukett Average (N=3) ± SD | 1602 ± 379 | 9,74 ± 0,13 | 2,9 |  |
