# Supplementary material for: Cold-Adapted Viral Attenuation (CAVA): Highly Temperature Sensitive Polioviruses as Novel Vaccine Strains for a Next Generation Inactivated Poliovirus Vaccine
Source: PLoS Pathog. 2016 Mar 31;12(3):e1005483. doi: 10.1371/journal.ppat.1005483 (PMC4816566; doi:10.1371/journal.ppat.1005483)
Supplement: S5 Table — Protocol adapted from [69] briefly, crude harvests were clarified over a series of filters (Clarified virus samples) and subsequently purified by Cation exchange and Size exclusion chromatography (Purified virus samples). Forty μl of Quantifluor dye (2000x diluted in double distilled H2O; Promega) was added to all sample solutions and three replicates per sample were transferred to a 96-wells PCR plate at 50 μl/well. The plate was covered with an optical adhesive film and placed in the 7500 Fast Real-Time PCR System (Applied Biosystems). The machine was set to ramp from 30°C to 79°C, taking a fluorescence reading at every 0.5°C increase per 30 seconds. Temperature melting point (Tm) was determined by calculation of the inflection point (the intercept of the second derivative of the x-axis) of the raw fluorescence data. An unpaired t-test was performed to assess if the difference in Tm between the CAVA strains and the respective cIPV strain is significant (two-tailed, α = 0.05). P-values are shown for each combination and an asterisk (*) represents a significant difference. (DOCX) [file ppat.1005483.s009.docx]

| Virus | Melting Temperature (Tm °C±SD) | | | |  |
| --- | --- | --- | --- | --- | --- |
|  | Clarified Virus Samples (N=3) Purified Virus Samples (N=2) | | | |  |
| CAVA-1 Mahoney | 47.3 (±1.2) | p = 0.089 | 52.5 (±0.6) | p = 0.382 | |
| Mahoney | 50.8 (±2.4) |  | 52.1 (±0.2) |  |  |
| CAVA-2 MEF-1 | 43.5 (±0.8) | p < 0.001* | 43.8 (±0.6) | p = 0.003* | |
| MEF-1 | 51.8 (±0.2) |  | 48.3 (±0.5) |  |  |
| CAVA-3 Saukett | 39.6 (±2.4) | p = 0.007* | 43.4 (±0.1) | p = 0.003* | |
| Saukett | 46.7 (±0.4) |  | 45.8 (±0.6) |  |  |
